# Hydrolase–like catalysis and structural resolution of natural products by a metal–organic framework

**DOI:** 10.1038/s41467-020-16699-3

**Published:** 2020-06-17

**Authors:** Marta Mon, Rosaria Bruno, Sergio Sanz-Navarro, Cristina Negro, Jesús Ferrando-Soria, Lucia Bartella, Leonardo Di Donna, Mario Prejanò, Tiziana Marino, Antonio Leyva-Pérez, Donatella Armentano, Emilio Pardo

**Affiliations:** 1 Instituto de Ciencia Molecular (ICMol), Universidad de Valencia, 46980 Paterna, Valencia Spain; 2 Dipartimento di Chimica e Tecnologie Chimiche (CTC), Università Della Calabria, 87036 Rende, Cosenza Italy; 3 Instituto de Tecnología Química (UPV–CSIC), Universitat Politècnica de València–Consejo Superior de Investigaciones Científicas, Avenida de los Naranjos s/n, 46022 Valencia, Spain

**Keywords:** Biocatalysis, Metal-organic frameworks, Metal-organic frameworks

## Abstract

The exact chemical structure of non–crystallising natural products is still one of the main challenges in Natural Sciences. Despite tremendous advances in total synthesis, the absolute structural determination of a myriad of natural products with very sensitive chemical functionalities remains undone. Here, we show that a metal–organic framework (MOF) with alcohol–containing arms and adsorbed water, enables selective hydrolysis of glycosyl bonds, supramolecular order with the so–formed chiral fragments and absolute determination of the organic structure by single–crystal X–ray crystallography in a single operation. This combined strategy based on a biomimetic, cheap, robust and multigram available solid catalyst opens the door to determine the absolute configuration of ketal compounds regardless degradation sensitiveness, and also to design extremely–mild metal–free solid–catalysed processes without formal acid protons.

## Abstract (web summary)

Elucidation of the chemical structure of natural products constitutes one of the main challenges in Natural Sciences. Here, the authors show that an amino acid-based metal–organic framework exhibits hydrolase-like catalytic activity and crystallographic determination of the resulting products.

## Introduction

The absolute structural configuration of natural products has been historically verified by total synthesis^1^, either from commercial compounds or, more conveniently, from fragments of the compound after controlled degradation and re-synthesis. However, the later approach is often hampered by the sensitiveness of natural complex molecules. For instance, the glycosyl bond^1^ (–O–CR_2_–O–) is prevalent in natural products since glycosidase (hydrolase) enzymes are widespread in all domains of life to generate (and break) ketals with an extremely high selectivity, at neutral pH in water, by the combined action of some amino acid residues in the confined enzyme electrostatic pocket. However, classical synthetic chemistry operates under much harder conditions, by using formal acids (i.e., protons and Lewis metal cations) or bases (i.e., inorganic bases and amines), which are clearly incompatible with the outstanding structural richness and sensitive functionality of ketals in Nature, and severely hampers the absolute determination of natural product structures by simple chemical degradation^2,3^.

Microporous solids may mimic enzymes with their active catalytic species in an electrostatic confined space^4,5^. Indeed, simple microporous aluminosilicates are good catalysts for ketal deprotection^6^, but they show low selectivity towards other acid sensitive functional groups, since the catalytic activity comes from acid protons associated with the solid network^7^. Early observations in microporous pure silicates showed that densely packed and interacting Si–OH groups, called silanol nests, naturally generate an acid site for catalysis without the participation of a formal proton^8,9^, however, the concept could not be extended to the organic functionalities present in enzymes, such as alcohols, since alcohols tend to generate either alkoxides^4^ or carbocationic species^10^ rather than acid sites, unless water^11^ or acetic acid^12^ are co-added. These precedents severely preclude simple alumisolicates to be used as catalase-like catalysts^13^ with extremely mild, bifunctional acid/base sites.

Metal–organic frameworks (MOFs)^14^ are porous crystalline materials amenable to single-crystal X-ray crystallography^15^ (SCXRD), with a great synthetic control of their high-dimensional architectures and concomitant porosity by a fine tuning of the functionalities decorating their channels using both pre- or post-synthetic^16,17^ methods. Indeed, their thrilling host–guest chemistry has led to the selective incorporation of gases, solvents, small molecules or more complex molecular systems^18,19^. Besides, advances like the crystalline sponge method^20^, developed by Fujita’s group, allows the absolute determination of organic molecules within the MOF framework^21–23^. Thus, seems plausible to go one-step further for the development of novel families of MOFs, specifically designed to combine the catalytic in-situ formation^1,24–28^, capture^29–31^, organization^19,32^ and retention of very sensitive unknown organic species within their functional channels^33^.

**Fig. 1:**
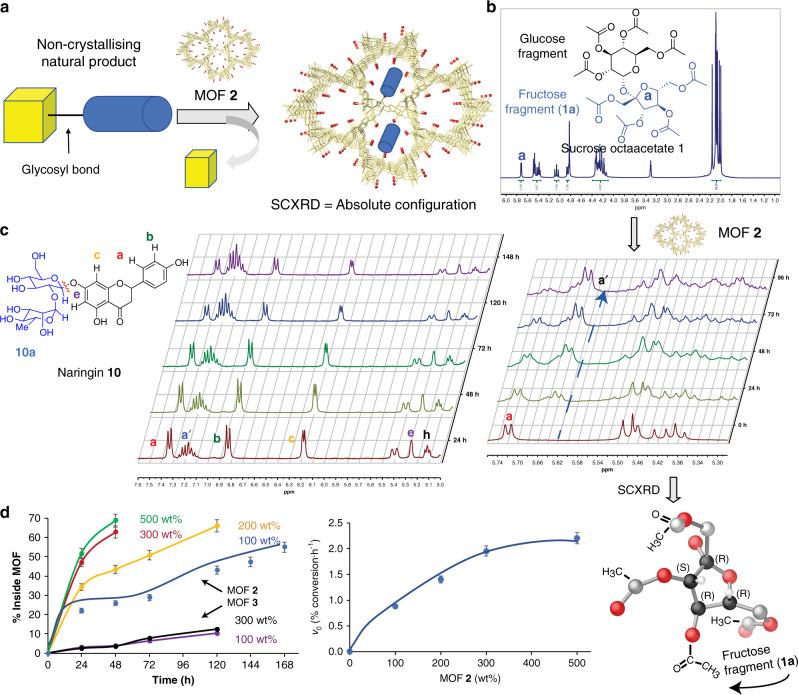
MOF-catalyzed selective hydrolysis of glycosyl bonds of sucrose 1. **a** Schematic representation of the one-pot selective hydrolysis/adsorption/crystal resolution of a natural product within a MOF. **b** ^1^H NMR spectra of sucrose **1** before and after selective hydrolysis with MOF **2**, and SCXRD resolution of fructose fragment **1a**. **c** Evolution with time of naringin **10** in CD_3_CN after reaction with MOF **2** at 60 °C, followed by ^1^H NMR. **d** Hydrolysis and incorporation of the alkyl part of naringin **10** into MOF **2** or MOF **3** with time for different amounts of MOFs (left) and initial rate as a function of the amount of MOF **2** employed (right), according to ^1^H NMR integrations. Reaction rates were measured with the initial points up to 30% conversion. Lines are a guide to the eye. Error bars account for a 5% uncertainty.

Herein, we report that a previously reported^30,31,34^ highly robust crystalline MOF-derived from the natural amino acid *l*-serine and whose micropores are densely decorated with methyl alcohol groups is capable to accommodate relatively big natural products, and performs, in a single operation, ketal deprotection and structural determination of sugars^1^ and flavonoids of known and unknown absolute configuration. After selectively incorporating, untouched, the fragment of unknown chirality into the MOF, the solid structure is resolved by SCXRD to give the absolute configuration of the adsorbed organic fragment (Fig. 1a) and, thus, of the natural product.

## Results

### Glycolysis of natural products of known structure

**Fig. 2:**
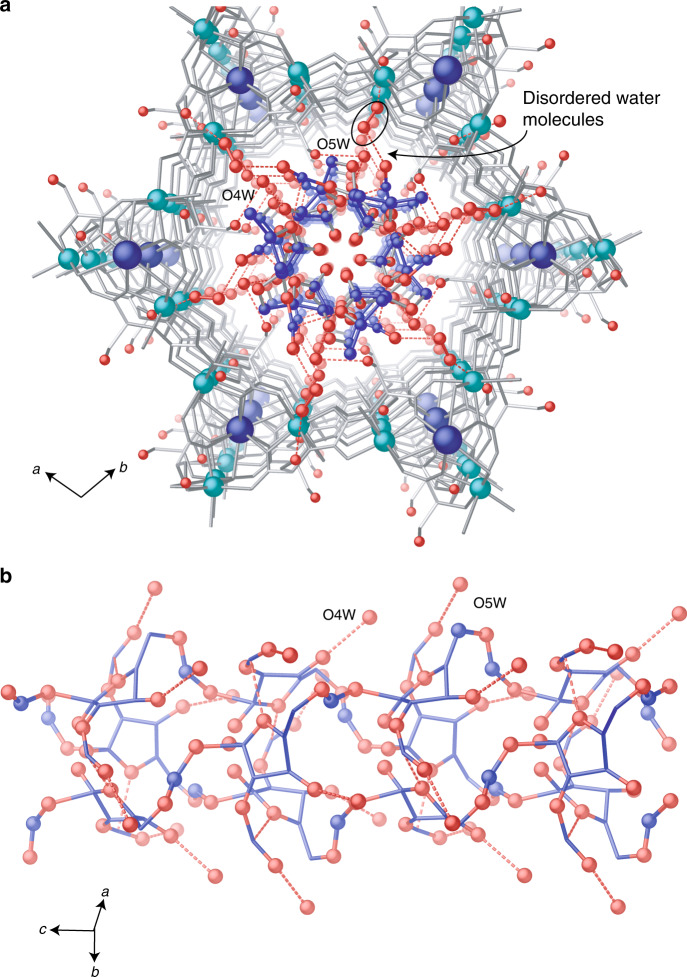
Crystal structure of 1a@2. **a** Perspective view along *c* crystallographic axis of a single channel underlining pores filled by guest molecules depicted as blue sticks with the only exception of oxygen atoms, depicted as red spheres. The H-bond interactions are depicted as red dashed lines. Disordered positions of lattice water molecules having a key role in host–guest interactions are highlighted (see details of refinement in Supplementary Methods). **b** Supramolecular chains of **1a** molecules packed in pores of **2** propagating along the direction of channels (all possible orientations are included). Carbon are represented by blue sticks whereas oxygen atoms of guest molecules and water molecules mediating host–guest interactions are represented by red spheres.

Figure 1b shows the proton nuclear magnetic resonance spectra (^1^H NMR) of a solution of sucrose octaacetate **1** (neat sucrose was not soluble under reaction conditions) recorded with time in the presence of the amino acid-based catalytic MOF **2**, which features hexagonal pores densely decorated with –OH groups, and with formula {Ca^II^Cu^II^_6_[(*S,S*)-serimox]_3_(OH)_2_(H_2_O)}. 39H_2_O (**2**) (where serimox^30,31,34^ = bis[(*S*)-serine]oxalyl diamide, see also Supplementary Fig. 1). Isostructural MOF alamox (**3**) of formula {Ca^II^Cu^II^_6_[(*S,S*)-alamox]_3_(OH)_2_(H_2_O)}. 32H_2_O (where alamox = bis[(*S*)-alanine]oxalyl diamide) without alcohol but only methyl pending groups was used for comparison. The results show that the NMR signal corresponding to the ketal linkage (a) and, in general, all the signals associated with one of the parts of ketal **1**, the fructose fragment **1a**, progressively disappears in solution in the presence of MOF **2**, while the glucose fragment **1b** remains. Conversely, no hydrolysis was observed with MOF **3**, lacking confined alcohol groups. Gas chromatography coupled to mass spectrometry (GC-MS) analyses confirmed the nearly exclusive presence of glucose fragments **1b** in solution after treatment with MOF **2** (Supplementary Fig. 2). SCXRD of a crystalline sample of MOF **2** reacted with **1** (see “Methods” and Supplementary Methods) rendered a new host–guest material with formula (**1a**)@{Ca^II^Cu^II^_6_[(*S,S*)-serimox]_3_(OH)_2_(H_2_O)}. 19H_2_O (**1a@2**) (where **1a** = 1,3,4,6-Tetra-*O*-acetylfructofuranoside), whose crystal structure as well as absolute configuration could be elucidated by SCXRD analysis (see Fig. 2 and Supplementary Table 1 and also an in-depth analysis of **1a@2** crystal structure in Supplementary Methods). The results show that the only fragment found inside MOF **2** is fructose **1a** and not any glucose derivative (Fig. 1b and Supplementary Figs. 3–6 and Fig. 2 and crystallographic section in SI for details). These fragments, well defined by furanose ring, that is completely assigned by electron density maps, reside in the pores of MOF **2** anchored by means of strong hydrogen bonds involving locked water molecules, which act as a bridge between serine moieties and fructose molecules. So, the alcohol groups show a prominent role, providing the suitable polar environment to host fructose molecules, effectively retained and organized within the pores.

In order to better visualize and also to determine the relative catalytic hydrolysis rate compared to alcohol dihydroxylation (a representative competitive reaction in natural product degradation), the benzaldehyde and cyclohexanone ketals **4** and **5**, and 2-phenyl-2-propanol **6**, were employed as substrates for ketal hydrolysis and the dehydroxylation reaction, respectively (Supplementary Fig. 7). The kinetic results showed the formation of carbonyl compounds **7** and **8** at a significant rate for MOF **2** (5 mol% of MOF structural units respect to the substrate), but not for MOF **3**. Remarkably, despite the formation of alkene **9** may be expected to be extremely easy with a highly stable benzylic carbocation intermediate, the hydrolysis rate is twice for the dehydroxylation of **6**. These results confirm that MOF **2** selectively catalyzes the hydrolysis of glycosyl bonds without significant degradation of alcohol-substituted chiral carbons.

To further validate the extremely mild solid-catalyzed glycosyl bond-breaking reaction, the commercially available flavonoid naringin **10** (Supplementary Fig. 8), with a more complex glycosyl structure, was treated with catalytic amounts of MOFs **2** and **3**. Figure 1c shows the progressive disappearance of the ^1^H NMR signals corresponding to the alkyl fragment **10a** respect to the aromatic part, the latter evolving to a different product in solution which, according to NMR and GC-MS, may be assigned to the oxidized quinone derivative. The spontaneous oxidation of the aromatic part in flavonoids, after losing the stabilizing glycosyl fragment, was expected according to the literature^10,35^. Figure 1d shows the increase in the hydrolysis rate of naringin **10** with MOF **2**, but not with MOF **3**, the latter in the same range that the hydrolysis rate without catalyst. All the results above, together, strongly support that MOF **2** selectively breaks glycosyl bonds in natural products and, concomitantly, adsorbs the so-formed alkyl chains, such as **1a** in sucrose and **10a** in naringin.

### Glycolysis and structural resolution of Brutieridin 11

**Fig. 3:**
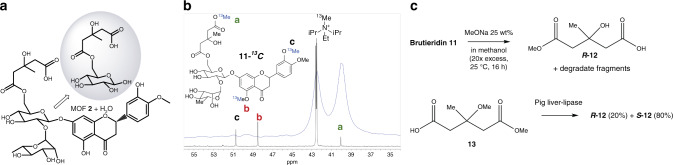
Hydrolysis of brutieridin 11. **a** Chemical structure of brutieridin **11**. Gray-colored ellipse highlights the fragment **11a**, which contains the chiral carbon and encapsulates within the MOF after the break of the glycosidic bonds. **b** Chemical structure and ^13^C NMR spectrum of **11-**^***13***^***C*** in solution (black line), and MAS solid ^13^C NMR spectrum of MOF **2** after hydrolysis of **11-**^***13***^***C*** (blue line). The isotopically labeled carbon atoms are lettered (**a**–**c**). **c** Hydrolisis of **11** with sodium methoxide to give chiral fragment **12**, and synthesis of **12** by enzymatic hydrolysis of diester **13**.

Aiming to further test our hypothesis, we studied a unique class of flavonoids that is found in bergamot fruit (Citrus Bergamia Risso et Poiteau) which, in addition to other citrus species such as naringin **10**, neohesperidin and neoeriocitrin, contains a relevant concentration of the anti-cholesterol agent 6-*O-*hydroxymethylglutaryl (HMG) ester derivative brutieridin **11** (Fig. 3a and Supplementary Figs. 9 and 10)^36–39^. Flavonoids are secondary metabolites widespread in Nature and involved in different metabolic processes, offering potential clinical alternatives to current treatments. However, so far, the challenging characterization of this kind of flavonoids has been carried out by the combination of High-performance liquid chromatography, MS, and NMR techniques, which have severe shortcomings for the proper identification of their chiral centers. For instance, brutieridin **11** has been isolated and identified with the formula hesperetin 7-(2′′-R-rhamnosyl-6′′-(3′′′′-hydroxy-3′′′′-methylglutaryl)-glucoside^36^, but all attempts to crystallize it, thus determining its crystal structure and unveiling its chiral nature, have been unsuccessful so far. Brutieridin **11** presents two glycosidic bonds and, beyond other sensitive functionalities, several secondary and tertiary chiral alcohols along its chemical structure. It is noteworthy the presence of an alcohol group flanked by two different carboxylic groups in beta position (fragment **11a**, Fig. 3a), an extremely sensitive chemical aggrupation prone to suffer degradation under both acid or basic conditions, since the tertiary alcohol dehydrates under acid conditions to generate a stable alkene conjugated to any of both carboxylic groups or, conversely, the alpha carbon to the carboxylic groups deprotonates under basic conditions to generate the same degraded products. These easy acid or base-triggered reactions, together with the interferences and potential side reactions caused by the phenol, ester, ketone and ether groups also present in brutieridin **11**, makes extremely difficult the selective degradation of this natural product by any classical deketalization method.

With the above data in mind, the hydrolysis of brutieridin **11** was attempted with MOF **2**. A combined ^1^H NMR, ultraviolet–visible spectrophotometry (UV–Vis), diffuse-reflectance UV–Vis spectrophotometry (DR–UV–Vis) and Fourier-transformed infrared spectroscopy (FT-IR) study was performed in order to follow concomitantly the fate of **11** in solution and within MOF **2**. ^1^H NMR results (Supplementary Fig. 11) show that the hydrolysis indeed occurs. The alkyl fragment **11a** progressively disappears from the solution, and the aromatic fragment transforms into a more symmetric aromatic molecule that persists in solution. This new aromatic molecule is the quinone fragment, according to UV–Vis and FT-IR (Supplementary Fig. 12) and also to GC-MS analysis (Supplementary Fig. 13), akin to what occurred during the hydrolysis of naringin **10**. DR–UV–Vis (Supplementary Fig. 14) and FT-IR measurements (Supplementary Fig. 15) of the solid MOF **2** after reaction reveal that the aromatic fragment does not incorporate into the pores, in line with the results observed for naringin **10**. To further confirm that the alkyl fragment **11a** and not the aromatic part accommodates inside the MOF pores, ^13^C isotopically labeled brutieridin (**11–**^***13***^***C***) was prepared and hydrolyzed with MOF **2** (Supplementary Fig. 16). Figure 3b shows that only the methoxy signal (a) assignable to the methyl ester of **11a** appears in the magic angle spinning (MAS) solid ^13^C NMR spectrum of MOF **2** after hydrolysis of **11–**^***13***^***C***. The two anisolic signals (b and c) of the aromatic fragment, present in the ^13^C NMR spectrum of **11–**^***13***^***C***, are not present. These results strongly support the selective incorporation of the chiral alkyl fragment **11a** to the crystalline MOF structure, yielding the novel hybrid material (**11a**)@{Ca^II^Cu^II^_6_[(*S,S*)-serimox]_3_(OH)_2_(H_2_O)}. 15H_2_O (**11a@2**), whose crystal structure as well as absolute configuration could now be elucidated by SCXRD analysis.

**Fig. 4:**
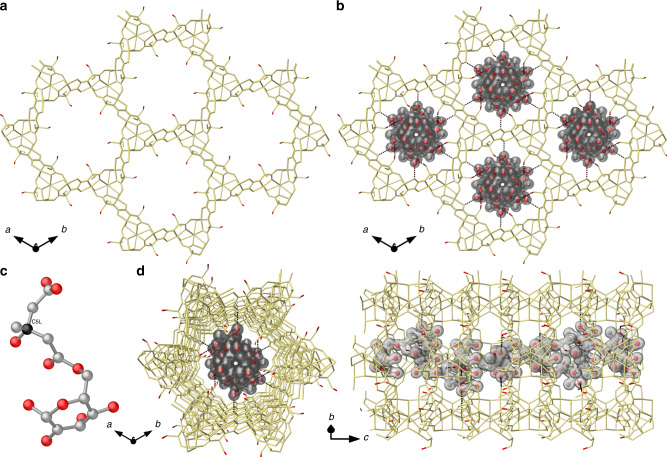
Crystal structure resolution of brutieridin 11. Views of the 3D open-framework of MOF **2** (**a**) and **11a@2** (**b**) along the *c*-axis (the crystallization water molecules are omitted for clarity). The 3D networks are depicted as gold sticks, with the only exception of serine residues oxygen atoms, which are represented as red sticks. **c** View of fragment of **11a** encapsulated within channels. **d** Top (left) and side (right) perspective views of a single channel of **11a@2**. Dashed lines represent hydrogen bonds between guest molecules involving also hydroxyl groups from the amino acid residue. The molecules of **11a** are shown as red (oxygen) and gray (carbon) stick/solid surfaces.

Figure 4 shows the structure of **11a@2**, determined by SCXRD, which confirms the preservation of the networks of **2** after guests’ capture. It is isomorphic to **2** crystallizing in the *P*6_3_ chiral space group of the hexagonal system and consists of a chiral honeycomb-like three-dimensional (3D) calcium(II)–copper(II) network, featuring functional hexagonal channels of ca. 0.9 nm as virtual diameter. The flexible hydroxyl (–OH) groups of the serine amino acid remain confined and stabilized by lattice water molecules, in the highly hydrophilic pores of the MOF (Fig. 4 and Supplementary Figs. 17–19). In these solvated nanospace, SCXRD underpinned **11a** molecules disclosing their configurations and locations, despite the persistent disorder. The uni-nodal **acs** six-connected net is built up from trans oxamidato-bridged dicopper(II) units, {Cu^II^_2_[(S,S)-serimox]} (Fig. 4), which act as linkers between the Ca^II^ ions through the carboxylate groups (Supplementary Fig. 1). Neighboring Cu^2+^ and Cu^2+^/Ca^2+^ ions are further interconnected by aqua/hydroxo groups (in a 1:2 statistical distribution) linked in a μ_3_ fashion (Supplementary Fig. 1c). Guest molecules of **11a** reside in the pores, packed via hydrogen bonds interactions, mediated by serine derivative arms (Fig. 4b–d and Supplementary Figs. 17–19). Moreover, intermolecular interactions of the chiral net of MOF **2** enabled that the chiral carbon of the HMG side chain of **11a** unveiled the *R* absolute configuration (Fig. 4c and Supplementary Fig. 20), with its bounded hydroxyl group directly interacting with methyl alcohol arm of the MOF (O5′···O1H_ser_ = 3.014(10) Å). The detailed structures showed **11a** arrangements (with a 1:3 statistical distribution, see Supplementary Methods for details and Supplementary Fig. 21), driven by the nature and size of the guest. In-depth analysis of the crystal structure reveals chiral **11a** molecules packed via a plethora of strong H-bonds, as expected for a polar molecule (Fig. 4 and Supplementary Figs. 18 and 19), involving hydroxyl serine derivative arms directly linked to hydroxyl groups attached to both sugar moiety or HMG side chain [O···O distances varying in the range 2.73(1)–3.01(1) Å] (Supplementary Fig. 19). Oxamate oxygen atoms from the coordination network assist with strong hydrogen bonds involving carboxyl of HMG [O_oxamate_···O_carboxyl_ distances of 2.69(1) Å]. The vastly solvated nano-confined space further supports the host–guest recognition process, mediating the interaction with the net by lattice water molecules acting as bridge between host and guests to reach the serine derivative arms [shortest O_11A_···O_W_ and related Ow···O_ser_ distances of 2.94(1) and 2.68(1) Å, respectively], also involving the innate flexible carboxyl terminus of HMG side chain. This is reminiscent of the interaction mechanisms found in glycosidases enzymes^40^.

Figure 3c shows that the controlled hydrolysis of brutieridin **11** with sodium methoxide gives the fragment derivative ***R***-**12** by chiral-phase GC analysis, together with significant amounts of the epimerized fragment and some other degraded fragments of the natural product, as determined with an independently synthesized, enantiomerically enriched sample of **12** obtained after enzymatic hydrolysis of diester **13** with pig liver estearase. Thus, one can say that the *R* configuration is thus obtained within MOF **2** and also by conventional hydrolysis. This result strongly supports the validity of the crystallization method to give the correct absolute configuration of unknown products by SCXRD.

### Mechanism of the MOF-catalyzed glycosyl hydrolysis

MOF **2** exposes, to outer molecules, a high number of densely packed alcohol groups, within a nanometer confined space, just as it occurs in the silanol nests of zeolites^8,9^. Thus, a particular acidification of adsorbed water, promoted by the cooperativeness between nearby methyl alcohols in MOF **2**, may occur. In order to discard potentially acidified water by interaction with the Cu^II^ atoms of the MOF network, the building block Cu_2_^II^[(S,S)-serimox] was also tested as a catalyst for glycosyl hydrolysis in wet conditions, and the results showed a catalytic activity of the free Cu^II^ species one order of magnitude lower than MOF **2** (Supplementary Fig. 22). Complementary, a filtration test showed that no leaching of active species occurs (Supplementary Fig. 23). Kinetic experiments with different amounts of reagents and catalyst (Supplementary Fig. 24) showed that the reaction orders for MOF **2**, ketal **4**, and water are 1, 1, and 0, respectively, thus giving a rate equation *v*_*0*_ = *k*_*exp*_[**2**][**4**]. The role of adsorbed water was further examined by dehydrating MOF **2** at 80 °C under high vacuum (10^−4^ mbar) during 16 h, and then performing the reaction with ketal **4** in anhydrous solvent, or adding 2 eq. of water to the reaction mixture at 60 °C. The results (Supplementary Fig. 25) show a 1/3 decrease of the hydrolysis rate compared to the original hydrated MOF **2**. These results support the catalytic action of water adsorbed in the alcohol network. Kinetic studies at different temperatures (25, 40, 60, and 80 °C) were carried out to calculate the activation energy of the glycolysis, which according to an Arrhenius plot was 15.0 kcal mol^−1^. The catalysis should occur mainly inside the MOF pores rather than on its external surface, as supported by the easier reactivity of small molecules, the saturation of the solid material with the bigger molecules and the X-ray data of the encapsulated fragments.

**Fig. 5:**
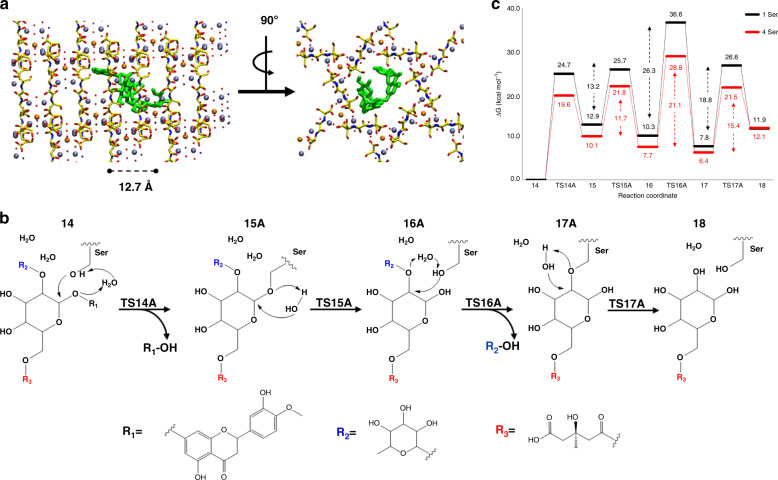
Theoretical mechanism for the MOF-catalyzed glycosyl hydrolysis. **a** The best docked pose selected on the basis of the applied geometrical and energetic filters. The studied catalytic mechanisms (**b**) followed in the hydrolysis reaction of **11** by MOF **2** and related PES (**c**) calculated at B3LYP-D3/6-311 + G(2d,2p)|UFF//B3LYP/6-31G(d)|UFF level of theory.

Computational studies were then performed to elucidate the possible mechanism of the glycosyl hydrolysis within MOF **2** (see Supplementary Methods), and the results are shown in Fig. 5. First, ten different complexes were generated by molecular recognition of **11** into the channel of MOF **2** (Supplementary Fig. 26). Based on the applied geometrical and energetic filters (see Supplementary Table 2), which evidenced that all the examined poses share a common orientation inside the channel of MOF **2**, the best docked pose was isolated (Fig. 5a) and used as starting structure in the Quantum Mechanics/Molecular Mechanics-Our own N-layered Integrated molecular Orbitals and Molecular mechanics (QM/MM–ONIOM) investigation (Supplementary Figs. 27 and 28). According to the calculations, the energetically favored mechanism for the hydrolysis of **11** within MOF **2** (see Fig. 5b, c) occurs by sequential cleavage of the two acetal C–O bonds, to release first the aromatic (R1) and then the cyclic aliphatic (R2) moieties, through the formation of intermediates bound to the serine residues (**15A** and **17A** in Fig. 5b). The potential energy surface (PES) related to the mechanism of Fig. 5b, black line of Fig. 5c, evidences that the hydrolysis of R1 (TS14A, 24.7 kcal mol^−1^) and R2 (TS16A, 26.3 kcal mol^−1^) occurs with comparable barriers. The energy barriers associated with an alternative mechanism where R2 is released before R1 (Supplementary Fig. 29) and also in the absence of MOF **2** (Supplementary Fig. 30) are significantly higher in both cases. Moreover, the calculations also highlight that the increased number of serine moieties (four versus one, see red line of Fig. 5c) leads to a lowering of the energy barriers (19.6 and 21.1 kcal mol^−1^, for TS14A and TS16A, respectively). This supports the synergic effect of the alcohol arms in the hydrophilic nano-confined space of the MOF and, extrapolating to the higher number of serine moieties present within MOF **2**, nicely points to the experimental value (15.0 kcal mol^−1^) obtained for the hydrolysis activation energy. It must be noticed that glycosyl hydrolysis occurs in Nature by two different mechanisms, either in one-step with acid/base sites and inversion of chirality (inverting glycoside hydrolases) or in two different steps with acid/nucleophile sites and retention of the starting chirality (retaining glycoside hydrolases). The catalytic action of MOF **2** perfectly lies on the retaining type hydrolases, with the combined action of mild acid sites (water) and nucleophile sites (alcohols) in a confined space.

## Discussion

An amino acid-derived MOF (**2**), densely decorated with methyl alcohol arms, is not only capable to hydrolyze glycosyl bonds like retaining hydrolase enzymes, without the participation of formal protons, but also to act as a vessel encapsulating the released chiral fragment and allowing the absolute structural determination of the natural product by SCXRD. The present results constitute a clear step forward on the use of MOFs in enzymatic catalysis^41,42^, where commonly their catalytic activity arises from preformed encapsulated active species. Also, let us anticipate that this bioinspired methodology with MOFs^41,43–47^ could have future application in the MOF-driven structural characterization^48,49^ of more natural product structures^15,50,51^.

## Methods

### Preparation of 1a@2

Well-formed hexagonal green prisms of **1a@2** ((**1a**)@{Ca^II^Cu^II^_6_[(*S,S*)-serimox]_3_(OH)_2_(H_2_O)}. 19H_2_O, where **1a** = 1,3,4,6-Tetra-*O*-acetylfructofuranoside), which were suitable for X-ray diffraction, were obtained by soaking crystals of **2** (ca. 5.0 mg) in saturated water solutions of sucrose octaacetate (**1**), for 48 h at temperature of 50 °C. The crystals were isolated by filtration on paper and air-dried. **1a@2:** Anal.: calcd for C_38_Cu_6_CaH_82_N_6_O_56_ (1940.42): C, 23.52; H, 4.26; N, 4.33%. Found: C, 23.50; H, 4.21; N, 4.36%. IR (KBr): *ν* = 1625, 1611, 1610 cm^−1^ (C=O).

### Preparation of 11a@2

Well-shaped hexagonal prisms of **11a@2** ((**11a**)@{Ca^II^Cu^II^_6_[(*S,S*)-serimox]_3_(OH)_2_(H_2_O)}. 15H_2_O, where **11a** = 6-*O*-(3′–hydroxy-3′-methylglutaryl)-glucopyranose)), suitable for SCXRD, could be obtained by soaking crystals of **2** (which had been treated before through a solvent exchange process for a week, recharging fresh acetonitrile solvent daily) in a saturated acetonitrile solution containing hesperetin 7-(2′′-R-rhamnosyl-6′′-(3′′′′-hydroxy-3′′′′-methylglutaryl)-glucoside) (brutieridin **11**) during two weeks. After this period, crystals were isolated by filtration and air-dried. Anal.: calcd for C_36_Cu_6_CaH_74_N_6_O_52_ (1844.33): C, 23.44; H, 4.04; N, 4.56%. Found: C, 23.39; H, 4.01; N, 4.57%; IR (KBr): *ν* = 1637, 1613, 1608 cm^−1^ (C – O).

### Catalytic procedures

MOF **2** or MOF **3** (37.5 mg, 100 wt%) were placed in a 2 ml vial equipped with a magnetic stir bar, and the corresponding amount of CD_3_CN (0.75 ml) was added. Then, the corresponding amount of naringin **10** (37.5 mg) was added at room temperature. The mixture was sealed and magnetically stirred in a pre-heated oil bath at 60 °C. For kinetic experiments, individual reactions were placed for each point and after centrifugation, the supernatant of the mixture reaction was periodically taken and analyzed by NMR. The same procedure was followed for brutieridin **11**.

### Preparation of ^13^*C* isotopically labeled 11–^13^*C*

Brutieridin **11** (10 mg 0.013 mmol) was placed in a round-bottomed flask equipped with a magnetic stir bar, and CD_3_CN (2 ml). Then, *N*,*N*-Diisopropylethylamine (7 μl, 0.039 mmol) and ^13^CH_3_I (5 μl, 0.078 mmol) were added via syringe at 0 °C. The mixture was sealed and magnetically stirred at room temperature for 12 h. After that, the reaction mixture was analyzed by ^13^C NMR.

### Single-crystal X-ray diffraction

Crystal data for **1a@2** and **11a@2**: Hexagonal, space group *P*6_3_, *T* = 90(2) K, *Z* = 2; **1a@2**: C_38_Cu_6_CaH_82_N_6_O_56_, *a* = 17.7840(16) Å, *c* = 12.5090(14) Å, *V* = 3426.2(7) Å^3^, *σ* = 1.881 g cm^3^, µ (mm^−1^) = 2.031; Absolute structure parameter (Flack) of 0.12(2). **11a@2**: C_36_Cu_6_CaH_74_N_6_O_52_, *a* = 17.9667(15) Å, *c* = 12.6886(12) Å, *V* = 3547.2(7) Å^3^, *σ* =  1.727 g cm^3^, µ (mm^−1^) = 1.953. Absolute structure parameter (Flack) of 0.13(2). Further details can be found in the Supplementary Information.

### Computational details

The solved X-ray structure of **MOF 2** has been adopted as starting point in the computational investigation. The molecular recognition has been applied to dock substrate **11** into **MOF 2**, generating ten different poses. The best docked pose has been undertaken to QMMM investigations. The same computational protocol has been previously applied in recent works. Further detailed description of the used methods is given in Supplementary Methods.

## Supplementary information

Supplementary Information.

## Data Availability

The authors declare that all the data supporting the findings of this work are available within the article and its Supplementary Information files or from the corresponding author upon request. The X-ray crystallographic data reported in this study (Figs. 1, 2, and 4, Supplementary Figs. 1 and 3–6 and Supplementary Table 1) have been deposited at the Cambridge Crystallographic Data Center (CCDC), under deposition numbers 1985884 (**1a@2**) and 1985885 (**11a@2**). These data can be obtained free of charge from The Cambridge Crystallographic Data Center via http://www.ccdc.cam.ac.uk/data_request/cif.
