# Publisher Correction: Hydrolase–like catalysis and structural resolution of natural products by a metal–organic framework

**DOI:** 10.1038/s41467-020-17170-z

**Published:** 2020-07-08

**Authors:** Marta Mon, Rosaria Bruno, Sergio Sanz-Navarro, Cristina Negro, Jesús Ferrando-Soria, Lucia Bartella, Leonardo Di Donna, Mario Prejanò, Tiziana Marino, Antonio Leyva-Pérez, Donatella Armentano, Emilio Pardo

**Affiliations:** 1 Instituto de Ciencia Molecular (ICMol), Universidad de Valencia, 46980 Paterna, Valencia Spain; 2 Dipartimento di Chimica e Tecnologie Chimiche (CTC), Università Della Calabria, 87036 Rende, Cosenza Italy; 3 Instituto de Tecnología Química (UPV–CSIC), Universitat Politècnica de València–Consejo Superior de Investigaciones Científicas, Avenida de los Naranjos s/n, 46022 Valencia, Spain

**Keywords:** Biocatalysis, Metal-organic frameworks, Metal-organic frameworks

Correction to: *Nature Communications* 10.1038/s41467-020-16699-3, published online 17 June 2020.

The original version of this article did not acknowledge Antonio Leyva-Pérez as a corresponding author.

This has now been corrected in both the PDF and HTML versions of the article.

