# Supplementary material for: Hydrolase–like catalysis and structural resolution of natural products by a metal–organic framework
Source: Nat Commun. 2020 Jun 17;11:3080. doi: 10.1038/s41467-020-16699-3 (PMC7300120; doi:10.1038/s41467-020-16699-3)
Supplement: Supplementary file 1 — Supplementary Information. [file 41467_2020_16699_MOESM1_ESM.pdf]

# Supplementary Information

## Hydrolase-like catalysis and structural resolution of natural products by a metal-organic framework

Mon et al.

## Supplementary Methods

**Materials.** All chemicals were of reagent grade quality. They were purchased from commercial sources and used as received. {$Cu_6Ca$[(S,S)-serimox]$_3$(OH)$_2$($H_2O$)} · 39$H_2O$ was prepared as reported previously.[1] Brutieridin was extracted and purified according to literature procedures.[2,3]

**Physical Techniques.** Elemental (C, H, N) analyses were performed at the Microanalytical Service of the Universitat de València. $^{1}$H, $^{13}$C and DEPT NMR spectra were recorded at room temperature on a Bruker AC 200 (200.1 MHz) spectrometer using the appropriate solvent containing TMS as an internal standard. FT–IR spectra were recorded on a Perkin-Elmer 882 spectrophotometer as KBr pellets. FT–IR spectra of the liquids were recorded on a Thermo Nicolet iS10 by impregnating the windows with a dichloromethane solution of the compound and leaving to evaporate before analysis. Absorption spectra were recorded on a Cary 300 UV–Vis spectrophotometer (UV0811M209, Varian). The thermogravimetric analyses were performed on crystalline samples under a dry $N_2$ atmosphere with a Mettler Toledo TGA/STDA 851$^{e}$ thermobalance operating at a heating rate of 10 ºC min$^{-1}$. All the products obtained were characterised by GC-MS, $^{1}$H-, $^{13}$C-NMR and DEPT. The characterisation given in the literature was used for comparison. Gas chromatographic analyses were performed in an instrument equipped with a 25 m capillary column of 5% phenylmethylsilicone. *N*-dodecane was used as an external standard. GC/MS analyses were performed on a spectrometer equipped with the same column as the GC and operated under the same conditions.

**X-ray Powder Diffraction Measurements.** Fresh polycrystalline samples of **2, 1a@2** and **11a@2** were introduced into 0.5 mm borosilicate capillaries prior to being mounted and aligned on an Empyrean PANalytical powder diffractometer, using Cu Kα

radiation ($\lambda$ = 1.54056 Å). For each sample, five repeated measurements were collected at room temperature (2$\theta$ = 2–60°) and merged in a single diffractogram.

**Gas Sorption.** $N_2$ adsorption isotherm at 77 K, of samples of **2** and **1a@2**, were acquired in a Micromeritics ASAP2020 instrument. Prior to the measurements, the samples were outgassed at 348 K under $N_2$ flow for 16 h.

**In–situ magic angle spinning–solid nuclear magnetic resonance (MAS–NMR) experiment.** The $^{13}$C solid–state NMR spectrum of MOF **2** after reacting with a solution of brutieridin **11-*$^{13}$C*** in $CD_3CN$ at 60 ºC for 3 days, filtering off and drying under vacuum, was recorded at room temperature with a Bruker AVIII HD 400 WB spectrometer. The glass insert was fitted into 7 mm rotors and were spun at 5 kHz in a Bruker BL7 probe. $^{13}$C CP/MAS NMR spectra were recorded with proton decoupling, with $^{1}$H 90° pulse length of 5 μs, and a recycle delay of 3s.

**Preparation of (1a)@{Ca$^{II}$Cu$^{II}_6$[(*S*,*S*)-serimox]$_3$(OH)$_2$(H$_2$O)} · 19H$_2$O (1a@2)** where **1a** = 1,3,4,6-Tetra-O-acetylfructofuranoside. Well-formed hexagonal green prisms of **1a@2**, which were suitable for X-ray diffraction, were obtained by soaking crystals of **2** (*ca.* 5.0 mg) in saturated water solutions of sucrose octaacetate (**1**), for 48 hours at temperature of 50 °C. The crystals were isolated by filtration on paper and air-dried. **1a@2:** Anal.: calcd for $C_{38}Cu_6CaH_{82}N_6O_{56}$ (1940.42): C, 23.52; H, 4.26; N, 4.33%. Found: C, 23.50; H, 4.21; N, 4.36%. IR (KBr): $\nu$ = 1625, 1611, 1610 cm$^{-1}$ (C=O).

**Preparation of (11a)@{Ca$^{II}$Cu$^{II}_6$[(*S*,*S*)-serimox]$_3$(OH)$_2$(H$_2$O)} · 15H$_2$O (11a@2)** where **11a** = 6-*O*-(3′-hydroxy-3′-methylglutaryl)-glucopyranose): Well-shaped hexagonal prisms of **1a@2**, suitable for SCXRD, could be obtained by soaking crystals of **2** (which had been treated before through a solvent exchange process for a week, recharging fresh acetonitrile solvent daily) in a saturated acetonitrile solution containing hesperetin 7-(2″-R-rhamnosyl-6″-(3″″-hydroxy-3″″-methylglutaryl)-glucoside)

(brutieridin **11**) during two weeks. After this period, crystals were isolated by filtration and air-dried. Anal.: calcd for $C_{36}Cu_6CaH_{74}N_6O_{52}$ (1844.33): C, 23.44; H, 4.04; N, 4.56%. Found: C, 23.39; H, 4.01; N, 4.57%; IR (KBr): $\nu$ = 1637, 1613, 1608 $cm^{-1}$ (C=O).

**X-ray crystallographic data collection and structure refinement.** Crystals of **1a@2** and **11a@2** adsorbates were selected and mounted on a MITIGEN holder in Paratone oil, and very quickly placed in a nitrogen stream cooled at 90 K to avoid the possible degradation upon desolvation. Diffraction data were collected on a Bruker-Nonius X8APEXII CCD area detector diffractometer using graphite-monochromated Mo-$K_{\alpha}$ radiation ($\lambda$ = 0.71073 Å). The data were processed through the SAINT[4] reduction and SADABS[5] multi-scan absorption software. The structure was solved with the SHELXS structure solution program, using the Patterson method. The model was refined with version 2018/3 of SHELXL against $F^2$ on all data by full-matrix least squares.[6,7]

As reported in the main text, even after a single-crystal to single-crystal process, the retained crystallinity of the 3D network of **2**, allowed the resolution of the crystal structures of **1a@2** and **11a@2**. In particular, in case of **1a@2** it must be underlined that crystals suffered also temperature of 50 °C for 48 hours. For that it is reasonable to expect somewhat mismatches from the routine expected diffraction patterns. Nevertheless, parameters indicating data quality (see also Supplementary Table 1) are as the following for **1a@2** and **11a@2**, respectively: resolution, 0.75−0.91 Å; $R_{int}$, 6.16%− 8.93%; $R$(all data), 8.18%−8.13%; Flack parameter, 0.12(2)-0.13(2); standard uncertainties of C−C bond lengths, 0.0220 Å. Within the limit of X-ray crystallography, we are confident that the structures found are consistent even more bearing in mind the multi-techniques approach that pervades characterizations in the whole paper.

In the refinement of both crystal structures all non-hydrogen atoms were refined anisotropically except some highly dynamically disordered atoms of serine moieties

pointing towards pores refined on two positions [O2H and O2H'] and guest molecules, together with lattice water molecules. Some restrains to make the refinement more efficient have been applied, for instance ADP components have been restrained to be similar to other related atoms, using SIMU 0.04 for disordered sections or EADP for group of atoms of the guest molecules expected to have essentially similar ADPs. In particular in **1a@2** the highly diffuse electron density suggests that C7C and C9C sites are disordered and shared with disordered solvent molecules, which were modelled as overlapped positions with EXYZ and EADP and their occupancy factors have been imposed accordingly (see Supplementary Fig. 5 for details). The high disorder detected is likewise at the origin of mismatch between expected and experimental values in some C-C bond lengths.

In **11a@2** atoms sharing the same site (in consequence of statistic disorder C1L with O2' and C8L with O7) have been refined as overlapped positions with EXYZ and EADP and their occupancy factors have been imposed accordingly. For both structures, the hydrogen atoms of the net were set in calculated position and refined isotropically using the riding model. Hydrogen atoms on the guest molecules and for solvent lattice molecules were neither found nor calculated. These molecules are expected to be severely disordered as a direct consequence of their high thermal motion and exhibit also statistic disorder [in **1a@2** O4W and O5W has been refined on two sites as O3W in **11@2**]. In general guest molecules are severely disordered, especially for the guest's fragments pointing towards the center of the pores where, undoubtedly, the degrees of freedom, related to diverse possible conformations, increase. It is well known that a crystal structure is the *spatial average*, representing all molecules, together with all their possible orientations averaged, in the crystal *via* only one-unit cell. In all cases, as the present one of as synthesized porous materials, where obviously not all unit cells are identical and a

variety of orientation are allowed, the description became more challenging. The occupancy of the guests in the pores was found via a free variable and later fixed at the converged occupancy. The use of some C-C and C-O bond lengths restraints of highly disordered atoms for guest molecules during the refinement has been reasonably imposed, as well as related to the expected and severe thermal motion, likely depending on the large size of the huge pores of the frameworks (SADI, DFIX, DANG, SIMU, and DELU). The guest molecules are also statistically disordered. In **1a@2** the highly diffuse electron density did not allow to model some terminal groups of acetate moieties, suggesting they are affected by very large thermal motion (see Supplementary Fig. 5 for details). In **11@2** the whole glucose fragment of the guest molecule resides on special positions and overlap on the symmetrically generated ones by the 6-fold rotation operation. A detail of kind of disorder related to guest molecules in **11a@2** crystal structure has been showed in Fig. S20-S21.

Overall the "Alert B" notifications found in the validation program CheckCIF are related either to intrinsic imperfections (as the presence of large outliers in the data set) quite normal for crystals that suffered a single-crystal to single-crystal process, or from short intermolecular contacts between water molecules and guest molecules or water molecules or guest molecules and the whole network are unavoidable due to the expected severe disorder of both solvent and guest molecules. Reflections that are affected by the beamstop or having $(I_{obs}-I_{calc})/\sigma > 10$ were omitted. The comments for the alerts are described in the CIFs using the validation response form (vrf).

A summary of the crystallographic data and structure refinement for **2**, **1a@2** and **11a@2** are given in Supplementary Table 1. CCDC Deposition Number are 1985884-1985885 for **1a@2** and **11a@2,** respectively.

The final geometrical calculations on free voids and the graphical manipulations were carried out with PLATON[8,9] implemented in WinGX,[10] and CRYSTAL MAKER[11] programs, respectively.

**In depth analysis of 1a@2 crystal structure.** The crystal structures of **1a@2** could be determined by single-crystal X-ray diffraction (SCXRD). It is isomorphs to **2** and crystallize in the $P6_3$ chiral space group of the hexagonal system. As previously reported, the structure of **2** consists of a chiral honeycomb-like 3D calcium(II)-copper(II) network featuring functional hexagonal channels, with virtual diameters of ca. 0.9 nm, where the flexible hydroxyl (–OH) groups of the serine amino acid remain confined, and stabilized by lattice water molecules, in the highly hydrophilic pores of the MOF (Supplementary Fig. 1). The **acs** six-connected net is built up from trans oxamidato-bridged dicopper(II) units, {$Cu^{II}_2$[(S,S)-serimox]} (Supplementary Fig. 1), which act as linkers between the $Ca^{II}$ ions through the carboxylate groups. Neighbouring $Cu^{2+}$ and $Cu^{2+}$/$Ca^{2+}$ ions are further interconnected by aqua/hydroxo groups (in a 1:2 statistical distribution) linked in a $\mu_3$ fashion. Despite the fructose terminal fragments were persistently disordered in the cage structures, we succeeded, within the limits of X-ray diffraction (XRD) crystallography, to get their possible configurations and locations by SCXRD, assigning all electron densities related to furanose ring. Further assignments of relatively strong electron densities around furanose core in the model improve the final refinements giving quite exhaustive snapshots of the incorporated molecules where only messy methyl groups and two of the four carbonyl moieties were not modelled. Undoubtedly, fructose fragments reside in the pores, thermally and statistically disordered, packed *via* hydrogen bonds interactions, water-mediated towards serine derivative arms (Supplementary Figs. 3-5).

The chiral centers of the furanose ring in the fragment **1a** have configuration 2*R*, 3*S*, 4*R*, 5*R* (Supplementary Fig. 6).

In depth analysis of **1a@2** crystal structure unveils chiral supramolecular chains of **1a** molecules developing along the direction of channels' propagation and packed *via* strong H-bonds involving hydroxyl groups interacting with lattice water molecules of the vastly solvated nano-confined space being anchored to the net by lattice water molecules acting as bridge to connect hydroxyl serine derivative arms [O···O distances varying in the range of 2.41- 2.98 Å] (Fig. S4). The severe disorder producing an overlap between the messy acetate terminal groups of the furanose ring and different conformations of adjacent molecules does not allow to spot any further detail. But meanwhile, suggests that the free rotation of especially methyl groups not so devoted to interact in hydrophilic environments and two of the four acetate moieties, most likely at the origin of such a high dynamic disorder, might reduce their aptitude to make any strong directional interaction. In fact, due to dynamic disorder, not all terminal –$CH_3COO$- groups have been modelled (see Supplementary Fig. 5) but an in depth analysis of organic molecule' configurations suggests that further O-H···O intermolecular interactions, between adjacent molecules, might ensure the cohesion of fructose molecules within pores.

**Catalytic procedures.** Reactions were performed in 2.0 ml vials equipped with a magnetic stirrer and closed with a steel cap having a rubber septum part to sample out, otherwise indicated.

*Hydrolysis and incorporation of the fructose part of sucrose octaacetate* ***1*** *into MOF* ***2****.* MOF **2** (31.5 mg, 100 wt%) were placed in a 2 ml vial equipped with a magnetic stir bar, and the corresponding amount of $CD_3OD$ (0,75 mL) was added. Then, the corresponding amount of sucrose octaacetate **1** (31.5 mg) was added at room temperature. The mixture was sealed and magnetically stirred in a pre–heated oil bath at 60 ºC. For kinetic

experiments, individual reactions were placed for each point and after centrifugation, the supernatant of the mixture reaction was periodically taken and analysed by NMR.

*<u>Hydrolysis of benzaldehyde and cyclohexanone ketals (**4** and **5**).</u>* MOF **2** or MOF **3** (50 mg, 100 wt%) were placed in a 2 ml vial equipped with a magnetic stir bar, and the corresponding amount of $CH_3CN$ (1 mL) was added. Then, ketal **4** (49 μl or 50 mg, 0.33 mmol) and water (12 μl, 0.66 mmol) were added via syringe at room temperature. The mixture was sealed and magnetically stirred in a pre–heated oil bath at 60 ºC for 4 h. For kinetic experiments, individual reactions were placed for each point and aliquots of 0.125 ml were periodically taken. After that, the reaction mixture was poured into dichloromethane (1 ml), *n*–dodecane (11 μl, 0.05 mmol) was added as an external standard, and the mixture was passed through a filter syringe and submitted to GC and GC–MS analysis. For NMR, solvent was removed under vacuum and the mixture was re–dissolved in $CDCl_3$, using TMS as an internal standard. Products were characterized by comparison with literature data. The same procedure was followed for ketal **5.**

*<u>Competitive dihydroxylation of 2-phenyl-2-propanol **8** vs hydrolysis of ketal **4**.</u>* MOF **2** (50 mg, 100 wt%) were placed in a 2 ml vial equipped with a magnetic stir bar, and the corresponding amount of $CH_3CN$ (1 mL) was added. Then, ketal **4** (49 μl or 50 mg, 0.33 mmol) and 2-phenyl-2-propanol **8** (47 μl or 46 mg, 0.34 mmol) were added via syringe at room temperature. The mixture was sealed and magnetically stirred in a pre–heated oil bath at 60 ºC for 4 h. For kinetic experiments, aliquots of 0.125 ml were periodically taken. After that, the reaction mixture was poured into dichloromethane (1 ml), *n*–dodecane (11 μl, 0.05 mmol) was added as an external standard, and the mixture was passed through a filter syringe and submitted to GC and GC–MS analysis.

**Synthesis of the chiral HMG fragment 12.** 4-Hydroxy-4-methyl-dihydro-pyran-2,6-dione (270 mg, 1.87 mmol) was slowly added over a suspension of 100 wt% of Amberlyst 15 (270 mg) in methanol (7 mL) in a 25 mL round bottomed flask equipped with a magnetic stir bar, at room temperature and under continuous stirring. Then, the mixture was left to reach 65 ºC during 3 h under stirring. An aliquot was dissolved in AcOEt (1 mL), the mixture was filtered through a 20 μm nylon filter and the resulting solution was analyzed by GC and GC–MS, confirming the obtention of dimethyl 3-hydroxy-3-methylpentanedioate **13**.

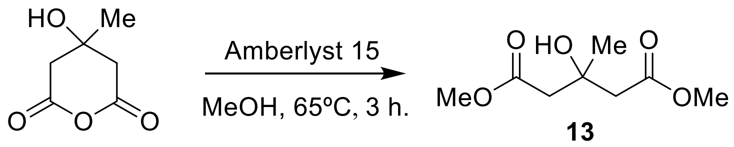


Phosphate buffer was prepared by dissolving 210 mg (1.2 mmol) of $K_2HPO_4$ in 12 mL of distilled water to give a solution having pH 8.9-9.0. To this buffer was added one vial (2.9 mg, 49 units) of pig liver esterase to give a solution having pH 8.6-8.7. The dimethyl 3-hydroxy-3-methylpentanedioate **13** obtanied in the previous step (100 mg, 0.53 mmol) was then added and the pH and time were recorded. The hydrolysis was allowed to progress until the pH had dropped to 7.2, corresponding to consumption of approximately 50% of the starting ester. The pH was then adjusted to 8.0 with NaOH 10% and the aqueous solution extracted with 3 x 15 mL of ether. The organic phase was then dried over $MgSO_4$ and filtered, and the solvent was evaporated to provide the unhydrolyzed ester fraction, which may be analyzed for enantiomeric purity without further purification. The aqueous phase was acidified to pH 2 with 1 N $H_2SO_4$ than again extracted with 3 x 15 mL. The organic phase was dried over $MgSO_4$ and filtered, and concentrated under vacuum. and the solvent evaporated to provide the hydrolyzed ester fraction as a mixture of both carboxylic acid chiral compounds enriched in the *S* enantiomer (ca. 80%) respect to the *R* enantiomer (ca. 20%). $[\alpha]^{24.2}$ = + 1.70 (MeOH).[12,13]

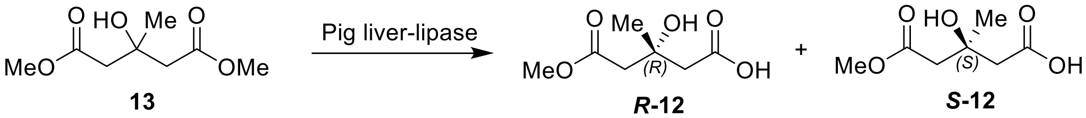

**Computational details.**

*Molecular Recognition*

The substrate **11** has been docked into a section of the crystal representing the minimal unit of MOF **2** (32.4 x 32.3 x 49.7 Å$^3$) adopted in the theoretical investigation. AutoDock Vina[14] code has been used in the molecular recognition and 10 output poses have been generated. Box centroid has been determined by a geometric center of the six serine moieties involved into the substrate-binding region and a box of 12.7 Å size for X, Y and 49.7 for Z was used for grid point generation. Each structure has been analyzed and the best docked pose with the lowest binding energy was selected for higher level DFT/MM mechanistic investigations.

*QMMM calculations*

The reaction mechanism has been investigated with one serine included in the high layer region (QM) and, in order to evaluate the effect of a higher number of serine on the energetic path, other three serines in proximity of brutieridin have been retained in the QM region. Furthermore, three water molecules, within 5 Å from the serine are present in the QM region while the remaining atoms of MOF and crystallographic waters belong to the lower layer (MM), as depicted in Supplementary Figs. 27-30. In all the cases the total charge of QM portion is equal to 0. Heavy atoms (Ca and Cu) have been fixed during the optimizations, in order to preserve structural stability and to avoid artificial movements of the architecture.

All the calculations were carried out by using the Gaussian 09 program.[15] The QM portions were treated with the B3LYP[16,17] hybrid density functional coupled to 6-31G(d) basis set. ONIOM scheme[18] was applied as the QM/MM method in the framework of electronic embedding scheme, in which the effects of the fixed MM charges are incorporated in the QM Hamiltonian.[19] The nature of optimized minima and transition

states on the potential energy surfaces were confirmed by the analysis of the corresponding Hessian matrices (one negative frequency for transitions states and no negative frequencies for minima). In order to improve the energies, single point calculations on the optimized structures were performed with the larger basis set 6-311+G(2d,2p). The final energies include the D3 dispersion correction[20] and zero-point-energy (ZPE) corrections were added to the final energies.

# Supplementary Tables

**Supplementary Table 1.** Summary of Crystallographic Data for **2**, **1a@2** and **11a@2**.

| Compound | **2**[a] | **1a@2** | **11a@2** |
|---|---|---|---|
| Formula | $C_{24}Cu_6CaH_{106}N_6O_{66}$ | $C_{38}Cu_6CaH_{82}N_6O_{56}$ | $C_{36}Cu_6CaH_{74}N_6O_{52}$ |
| $M$ (g mol$^{-1}$) | 1956.46 | 1940.41 | 1844.33 |
| λ (Å) | 0.71073 | 0.71073 | 0.71073 |
| Crystal system | Hexagonal | Hexagonal | Hexagonal |
| Space group | $P6_3$ | $P6_3$ | $P6_3$ |
| $a$ (Å) | 17.8904(16) | 17.7840(16) | 17.9667(15) |
| $c$ (Å) | 12.7999(13) | 12.5090(14) | 12.6886(12) |
| $V$ (Å$^3$) | 3547.9(7) | 3426.2(7) | 3547.2(7) |
| $Z$ | 2 | 2 | 2 |
| $\rho_{calc}$ (g cm$^{-3}$) | 1.831 | 1.881 | 1.727 |
| $\mu$ (mm$^{-1}$) | 1.972 | 2.031 | 1.953 |
| $T$ (K) | 90 | 90 | 90 |
| $\theta$ range for data collection (°) | 1.314-26.958 | 2.290 - 26.430 | 1.309-26.103 |
| Completeness to $\theta$ = 25.0 | 100% | 100% | 100% |
| Measured reflections | 76915 | 27207 | 70300 |
| Unique reflections (Rint) | 5149 (0.0145) | 4703(0.0616) | 4720 (0.0893) |
| Observed reflections [$I > 2\sigma(I)$] | 4687 | 3457 | 3657 |
| Goof | 1.099 | 1.042 | 1.062 |
| Absolute structure parameter (Flack) | 0.07(4) | 0.12(2) | 0.13(2) |
| $R^b$ [$I > 2\sigma(I)$] (all data) | 0.0410 (0.0474) | 0.0530 (0.0818) | 0.0608 (0.0813) |
| $wR^c$ [$I > 2\sigma(I)$] (all data) | 0.1184 (0.1284) | 0.1427 (0.1621) | 0.1754 (0.1935) |
| CCDC CODE | 1823991 | 1985885 | 1985885 |

[a] Crystallographic data of the previously reported MOF **2** (references 30, 31 and 34) is shown here for the sake of comparison with **1a@2** and **11a@2**. [b] $R = \sum(|F_o| - |F_c|)/\sum|F_o|$. [c] $wR = [\sum w(|F_o| - |F_c|)^2/\sum w|F_o|^2]^{1/2}$.

**Supplementary Table 2.** Results for AutoDock Vina scoring using brutieridine in presence of MOF **2** and average distance between C1 and C2 atoms of substrate and serines in direct proximity.

| | Binding Affinity (kcal mol$^{-1}$) | $O_{Ser}$-$C1_{Substrate}$ ($\bar{d} \pm \sigma$ Å) | $O_{Ser}$-$C2_{Substrate}$ ($\bar{d} \pm \sigma$ Å) |
|---|---|---|---|
| **Dock1** | -9.5 | 6.77 ± 1.78 | 6.46 ± 1.05 |
| **Dock2** | -9.5 | 6.75 ± 1.75 | 6.53 ± 0.91 |
| **Dock3** | -9.4 | 6.73 ± 1.72 | 6.43 ± 0.97 |
| **Dock4** | -9-4 | 6.79 ± 1.80 | 6.46 ± 1.16 |
| **Dock5** | -9.3 | 6.91 ± 2.13 | 6.64 ± 1.56 |
| **Dock6** | -9.2 | 6.75 ± 1.74 | 6.53 ± 1.11 |
| **Dock7** | -9.2 | 7.42 ± 2.38 | 6.62 ± 1.52 |
| **Dock8** | -9.1 | 7.06 ± 2.36 | 6.63 ± 1.71 |
| **Dock9** | -9.1 | 6.77 ± 1.97 | 6.69 ± 1.57 |
| **Dock10** | -9.1 | 7.02 ± 2.17 | 6.94± 2.19 |

## Supplementary Figures

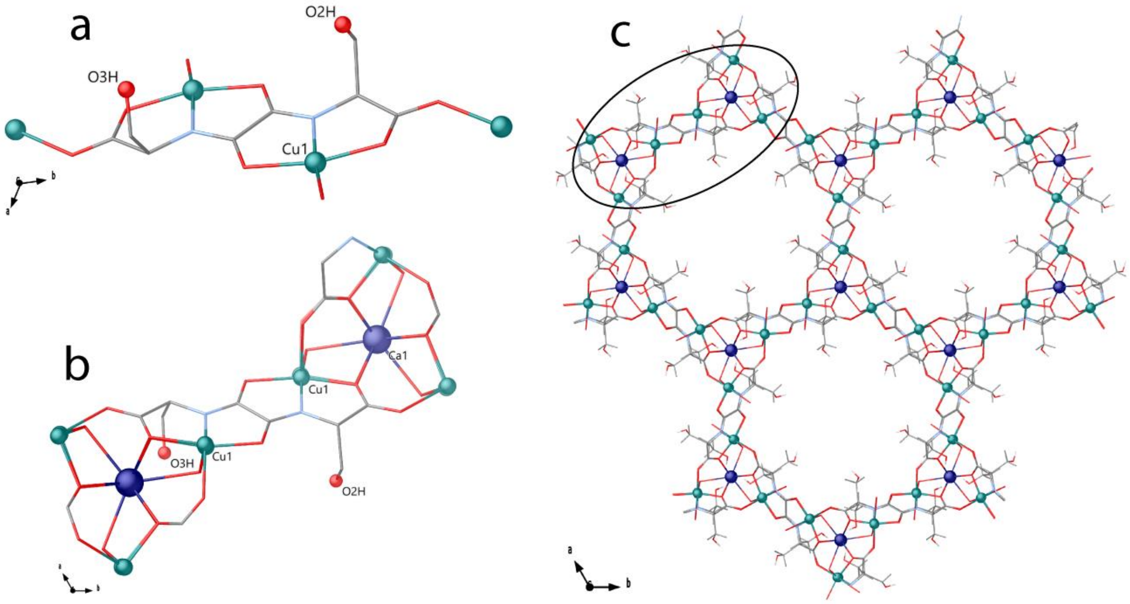


**Supplementary Fig. 1.** Views of a portion of X-ray crystal structure of **MOF 2**: (a) fragment of **2** showing the dianionic bis(hydroxo) dicopper(II) building blocks further connected (b) by $Ca^{2+}$ ions. (c) Views of a fragment of **2** in the *ab* plane. Copper, calcium and oxygen atoms of serine moieties are represented by green, blue and red spheres, respectively, whereas the ligands are depicted as sticks (carbon: gray, oxygen: red and nitrogen: light blue). Free water molecules residing in the pores are omitted for clarity.

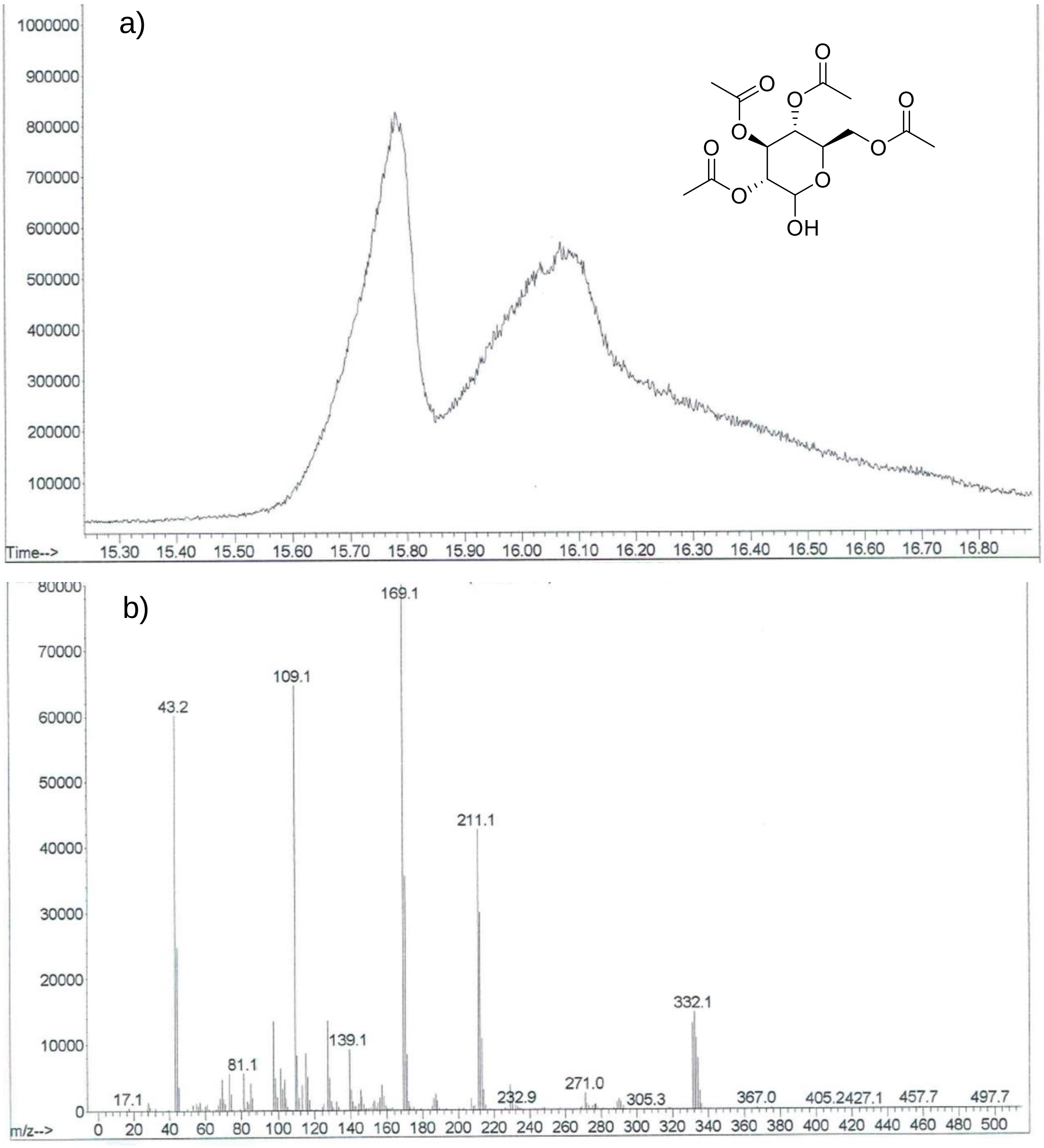


**Supplementary Fig. 2.** a) GC-MS of the glucose tetracetate derivative in solution, in both alpha and beta forms, as assessed by comparison with pure samples. b) The corresponding mass spectra of the dehydroxylated molecular ion ($M^{+\cdot}$= 331 u.m.a.) in different protonated forms.

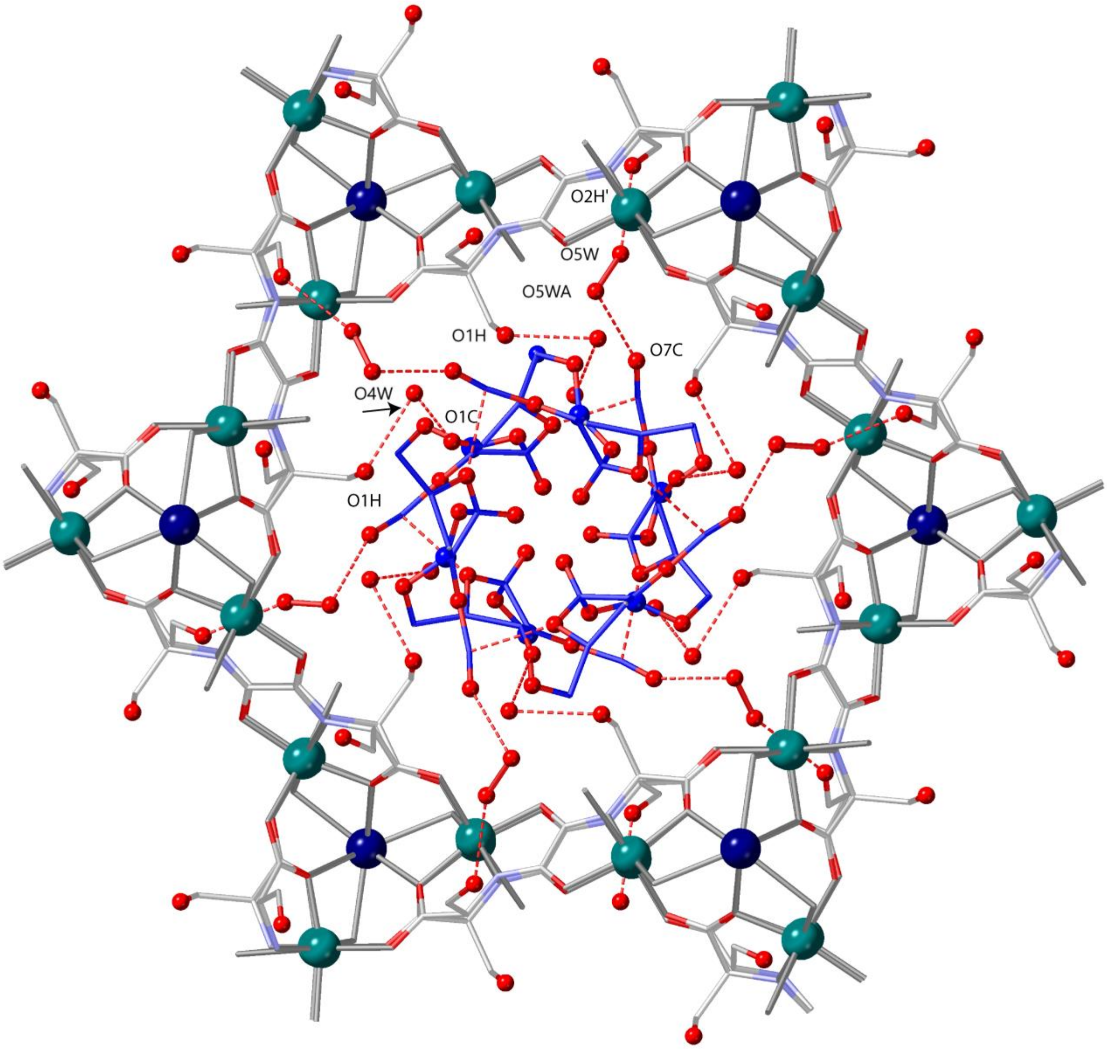


**Supplementary Fig. 3.** Crystal Structures of **1a@2**: Perspective view of a single channel in crystal structure of **1a@2** emphasizing pores filled by guest molecules (blue sticks with the only exception of oxygen atoms, which are depicted as red spheres). The H-bond interactions are depicted as red dashed lines. Continuous red lines underline double positions of water molecules having a key role in host-guest interactions. The only lattice water molecules depicted are those involved in the stabilization of the final serine residue configuration. The two crystallographically distinct serine arms, not so blocked, shows statistical disorder (see details of refinement in Crystallographic section).

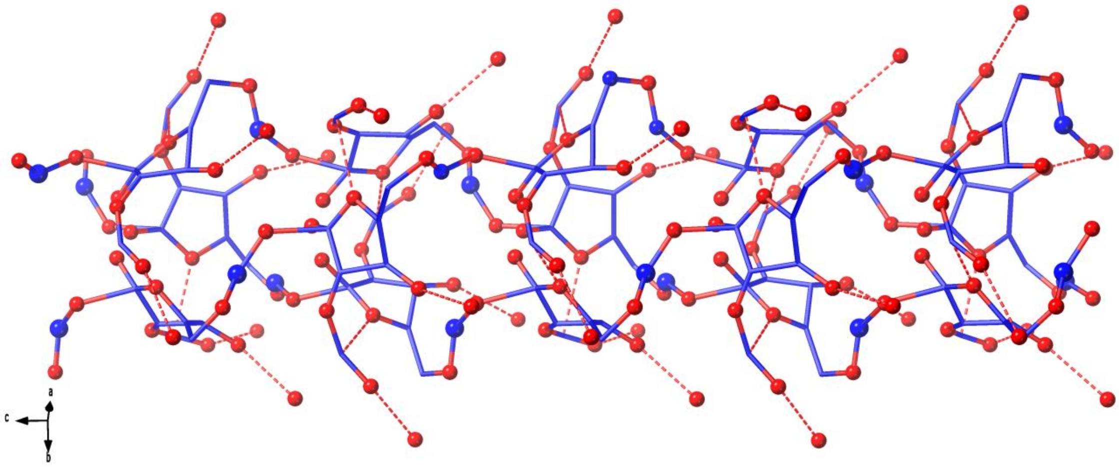


**Supplementary Fig. 4.** Crystal Structures of **1a@2**: Supramolecular *chains* of **1a** molecules packed propagating along the direction of channels (all possible orientations are included). Carbon are represented by blue sticks whereas oxygen atoms of guest' molecules and water molecules mediating interaction with the host **2** are represented by red spheres.

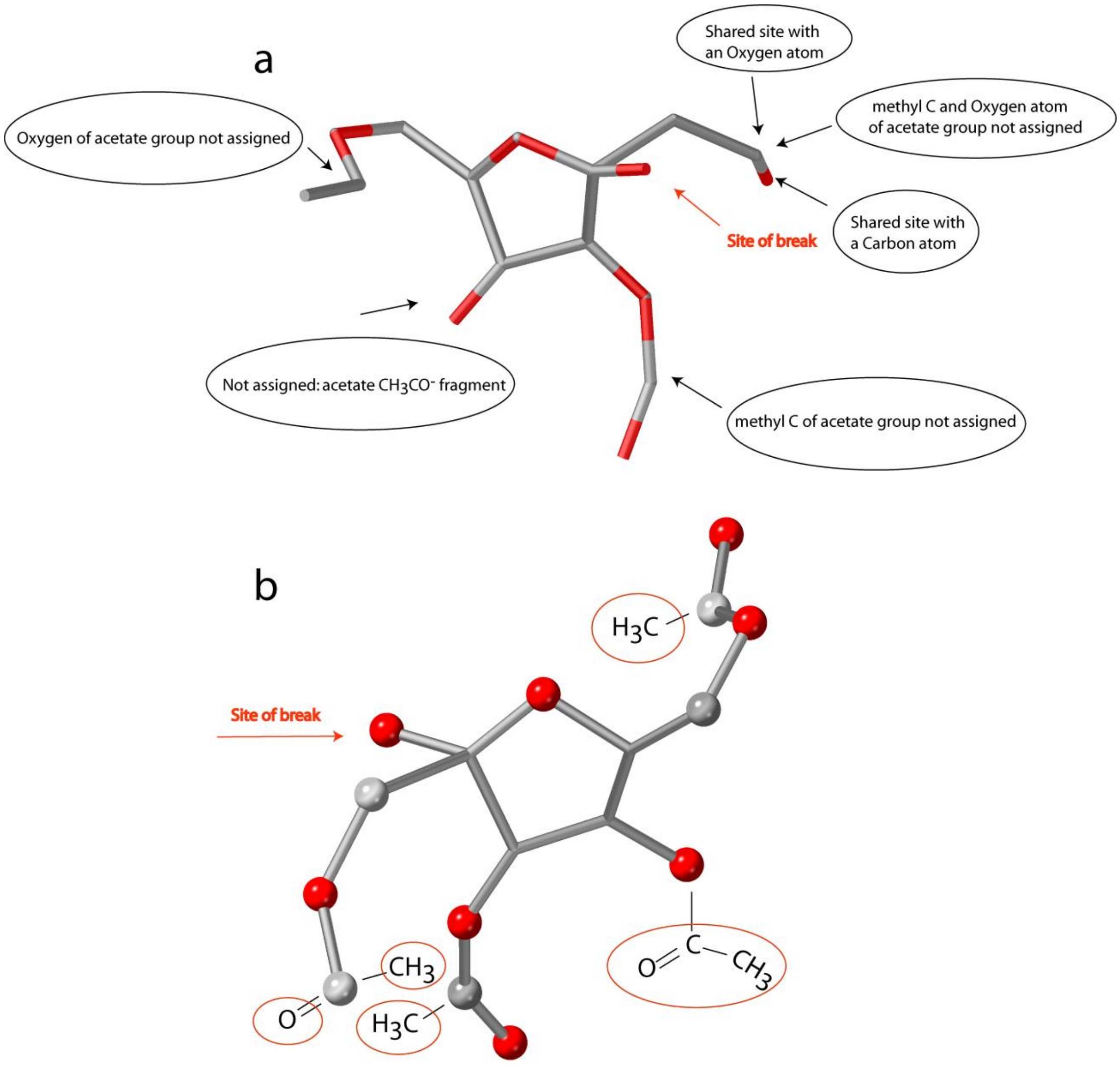


**Supplementary Fig. 5.** Crystal Structures of **1a@2**: a) Details of structural resolution of fructose moiety underlining sites shared for statistical disorder and b) groups of terminal atoms not found/defined from ΔF maps.

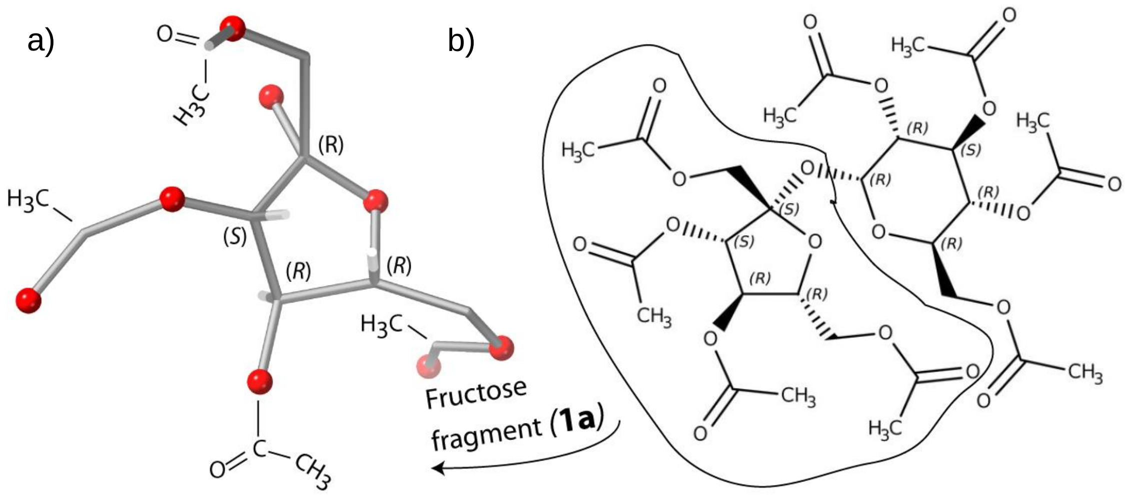


**Supplementary Fig. 6**. a) Absolute configuration in furanose ring of fructose fragment **1a** residing within pores of **2**; b) chemical structure of **1**, circled section represents fructose fragment **1a**.

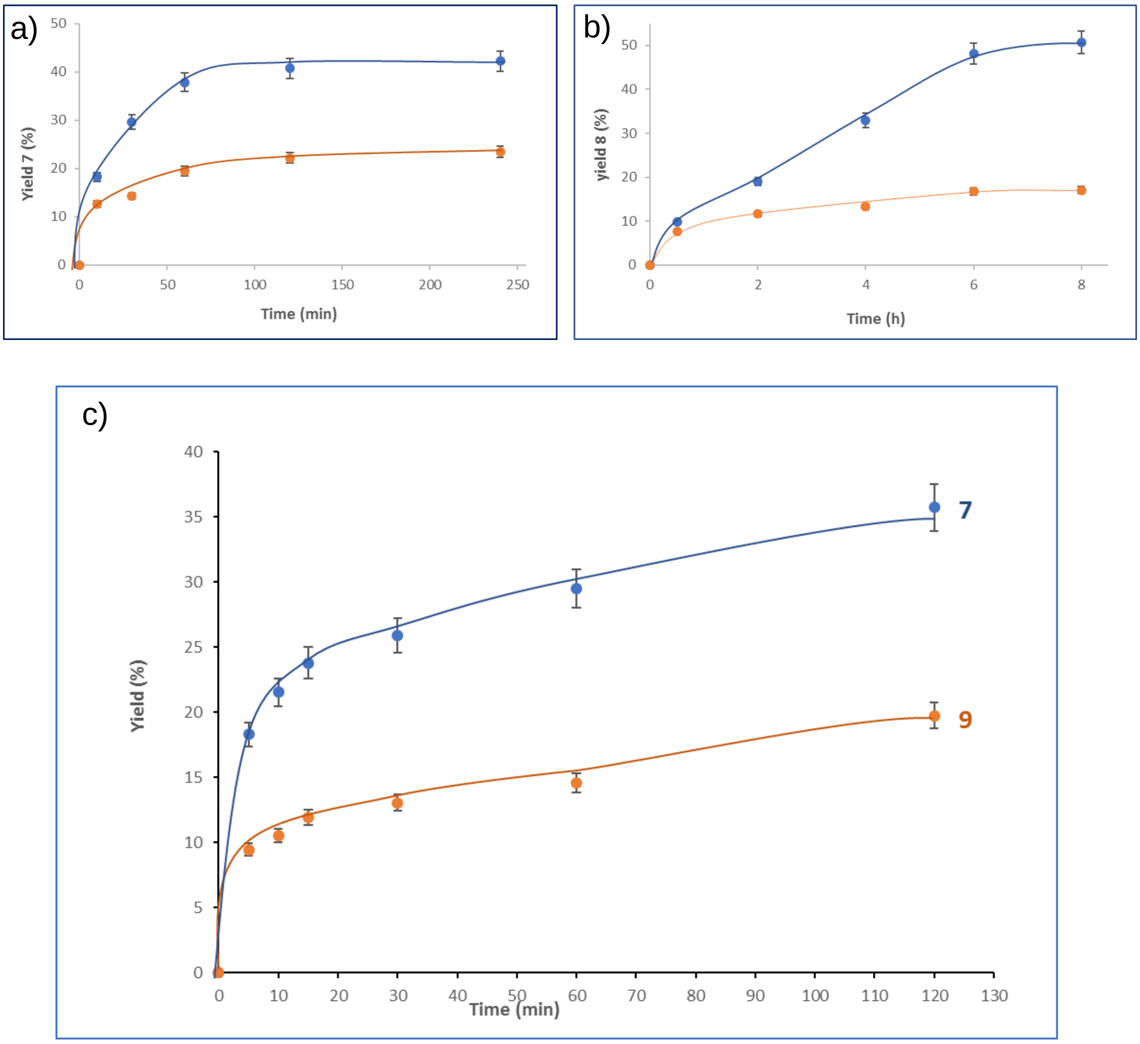


**Supplementary Fig. 7**. Kinetic results for the hydrolysis of a) benzaldehyde ketal **4** to benzaldehyde **7**, b) cyclohexanone ketal **5** to cyclohexanone **8**, and c) kinetic results for the competitive dehydroxylation of 2-phenyl-2-propanol **6** to alkene **9** *vs.* hydrolysis of benzaldehyde ketal **4**; in the presence of MOFs **2** (blue line) and **3** (orange line). Yields calculated by gas chromatography (GC) with *n*-dodecane as an external standard. Error bars account for a 5% uncertaintity. Lines are a guide to the eye.

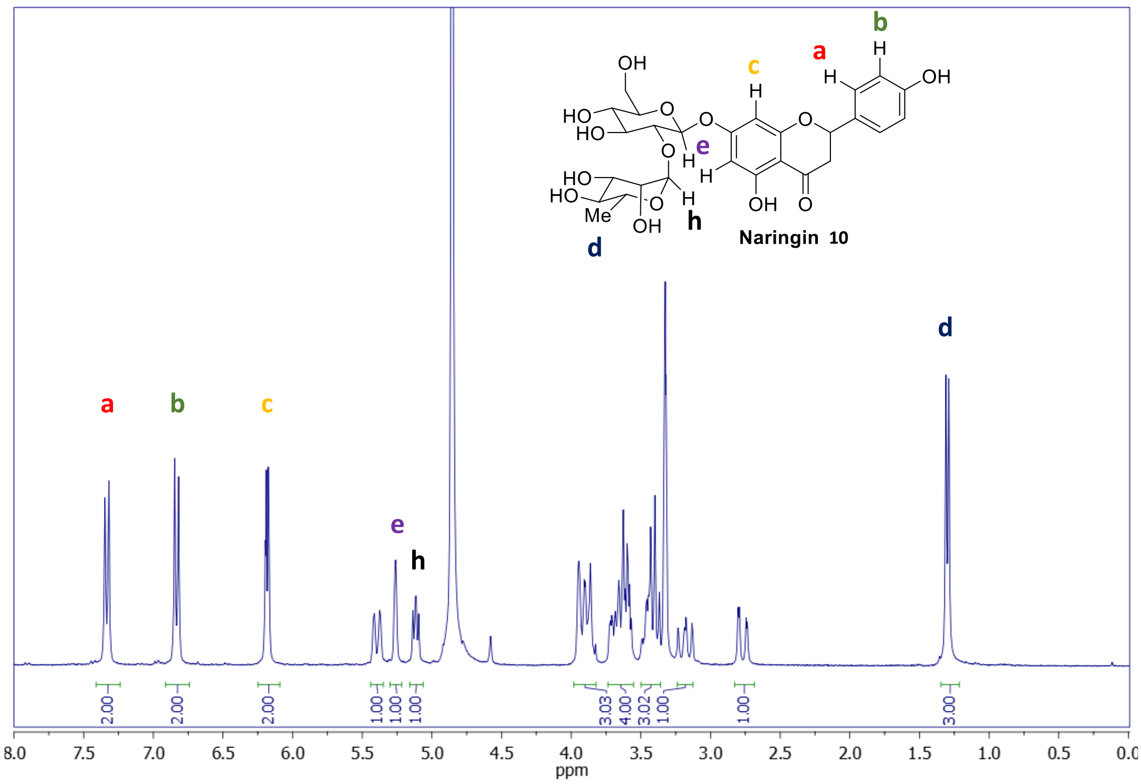


**Supplementary Fig. 8**. Chemical structure and $^{1}$H NMR, with assignations, of naringin **10**.

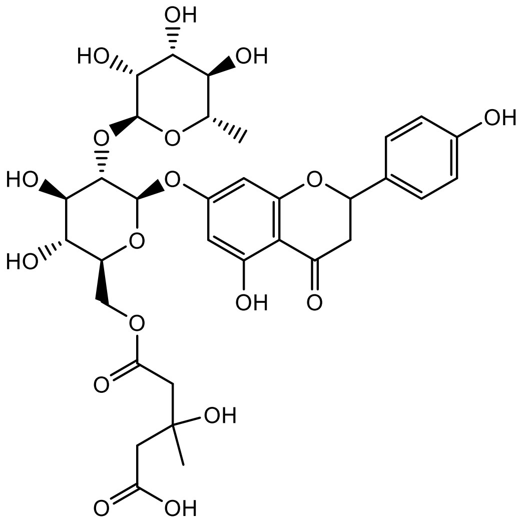

Melitidin

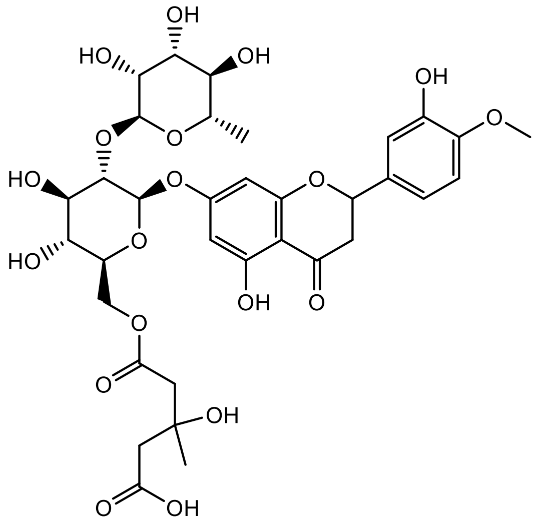

Brutieridin

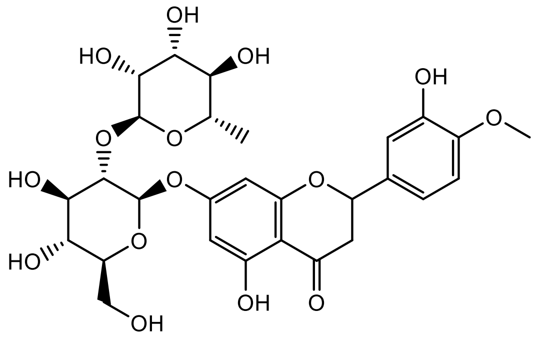

Neohesperidin

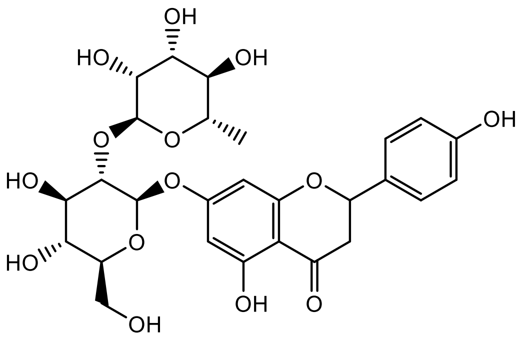

Naringin

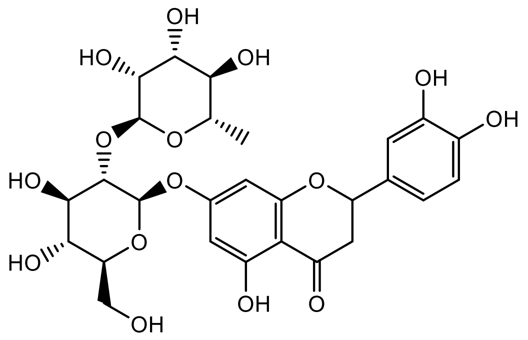

Neoeriocitrin

**Supplementary Fig. 9**. Chemical structure of the main flavonoids that are present in the bergamot phenolic fraction.

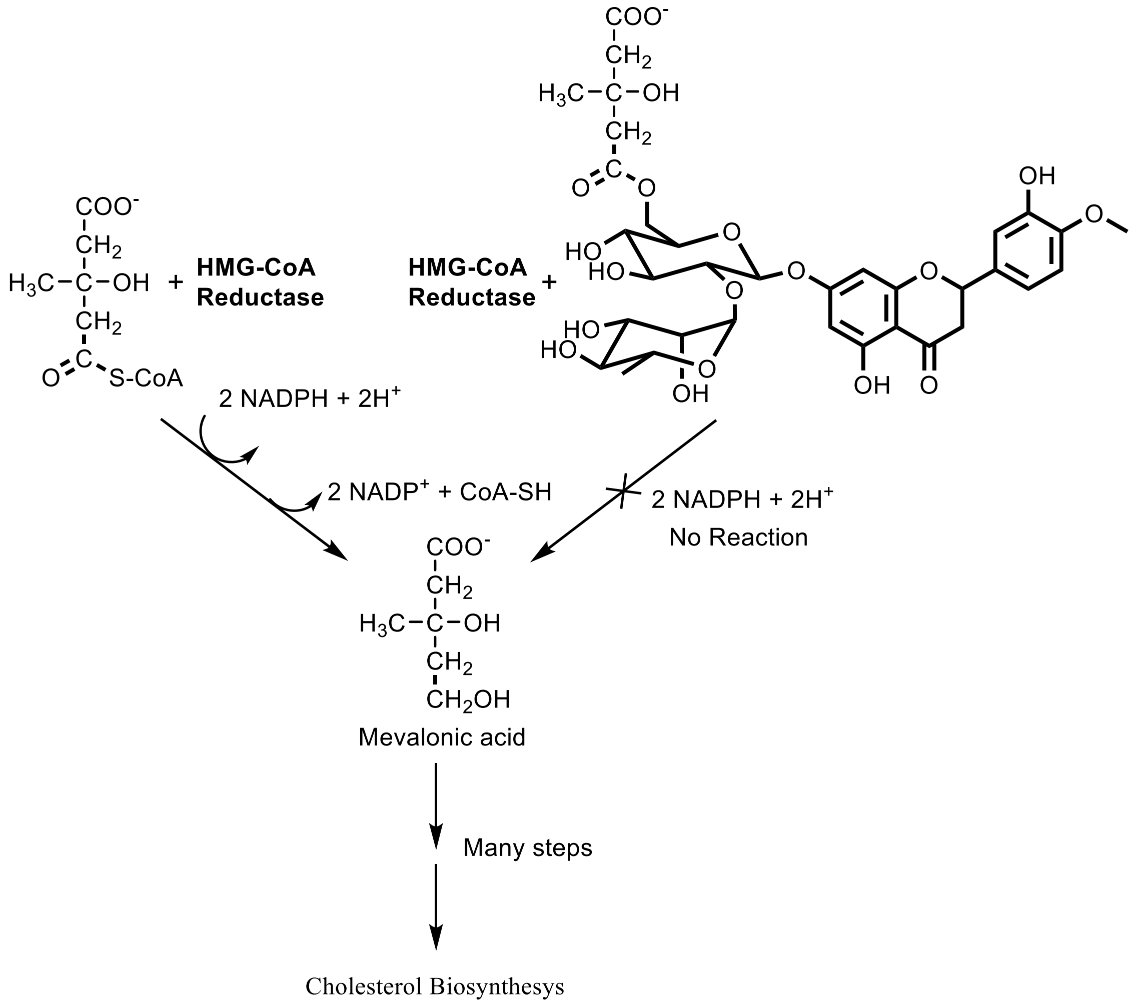


**Supplementary Fig. 10.** Inhibition of the reduction step to mevalonic acid by brutieridin in the cholesterol biosynthesis pathway.

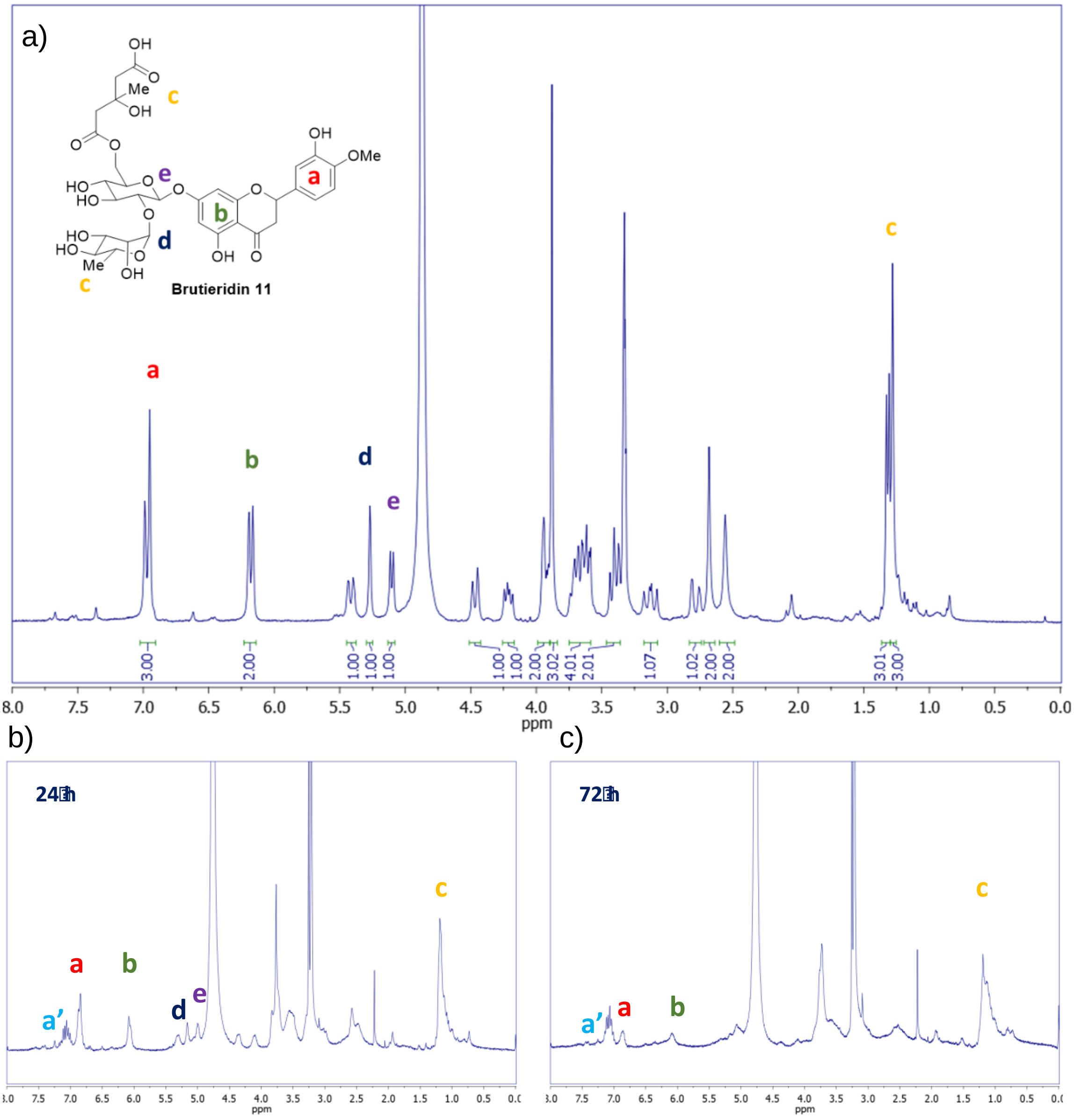


**Supplementary Fig. 11**. a) Chemical structure and $^{1}$H NMR, with assignations, of brutieridin **11**. b,c) Evolution with time in the presence of MOF **2** at 60 ºC, in $CD_3OD$. The a´ signal corresponds to the aromatic compound formed in solution after hydrolysis.

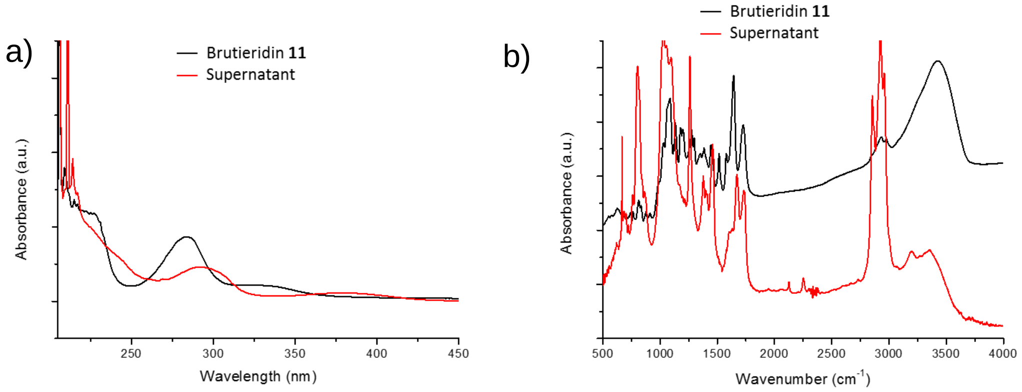


**Supplementary Fig. 12**. UV-vis (a) and FT-IR (b) spectra of a brutieridin **11** solution in MeOH before (black line) and after treatment with MOF **2** at 60 ºC (red line).

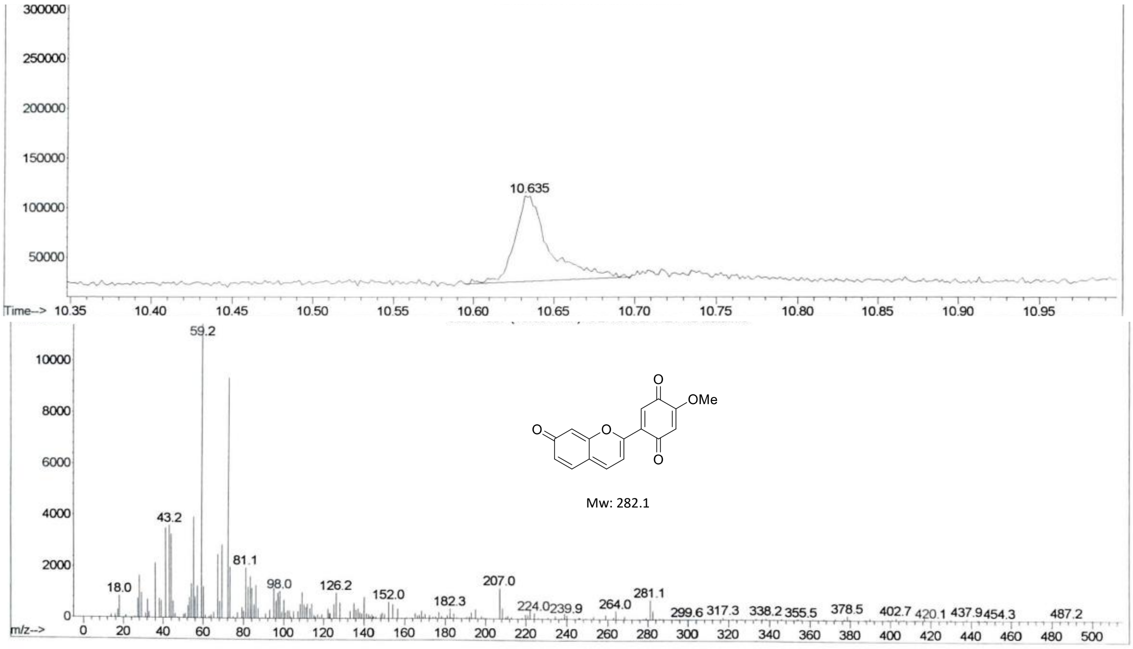


**Supplementary Fig. 13**. Mass spectrum of a brutieridin **11** solution in MeOH after treatment with MOF **2** at 60 ºC.

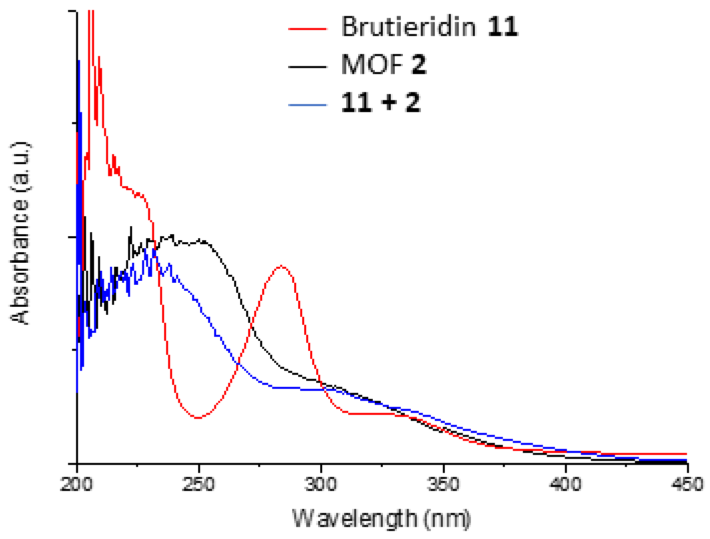


**Supplementary Fig. 14**. DR-UV-vis spectra of MOF **2** before (black line) and after (blue line) reacting with a solution of brutieridin **11** in MeOH at 60 ºC for 3 days, filtering off and drying under vacuum. For the sake of comparison, the UV-vis spectrum of **11** is also shown (red line).

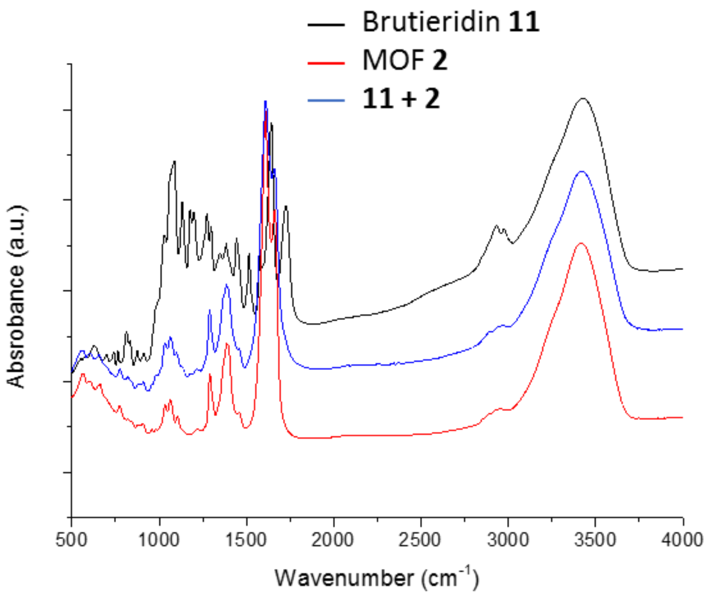


**Supplementary Fig. 15**. FT-IR spectra of MOF **2** before (red line) and after reacting with a solution of brutieridin **11** in MeOH at 60 °C for 3 days, filtering off and drying under vacuum (blue line). For the sake of comparison, the FT-IR spectrum of **11** is also shown (black line).

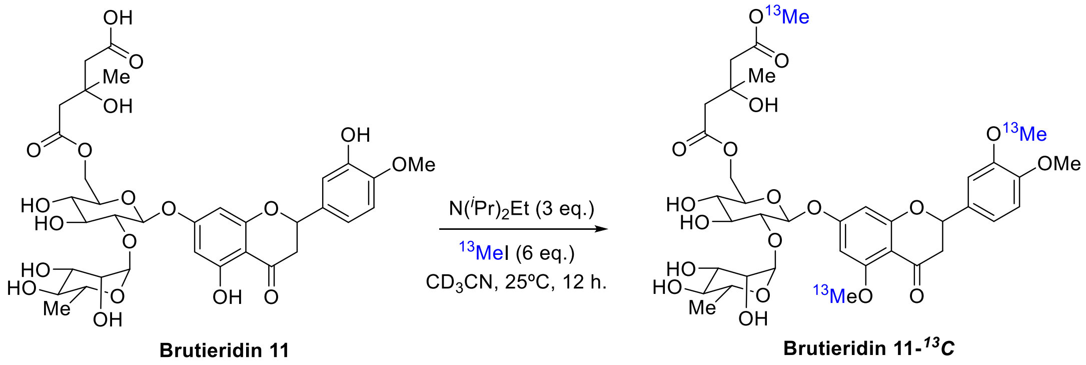


**Supplementary Fig. 16**. Preparation of *$^{13}$C* isotopically labelled brutieridin (**11-*$^{13}$C***).

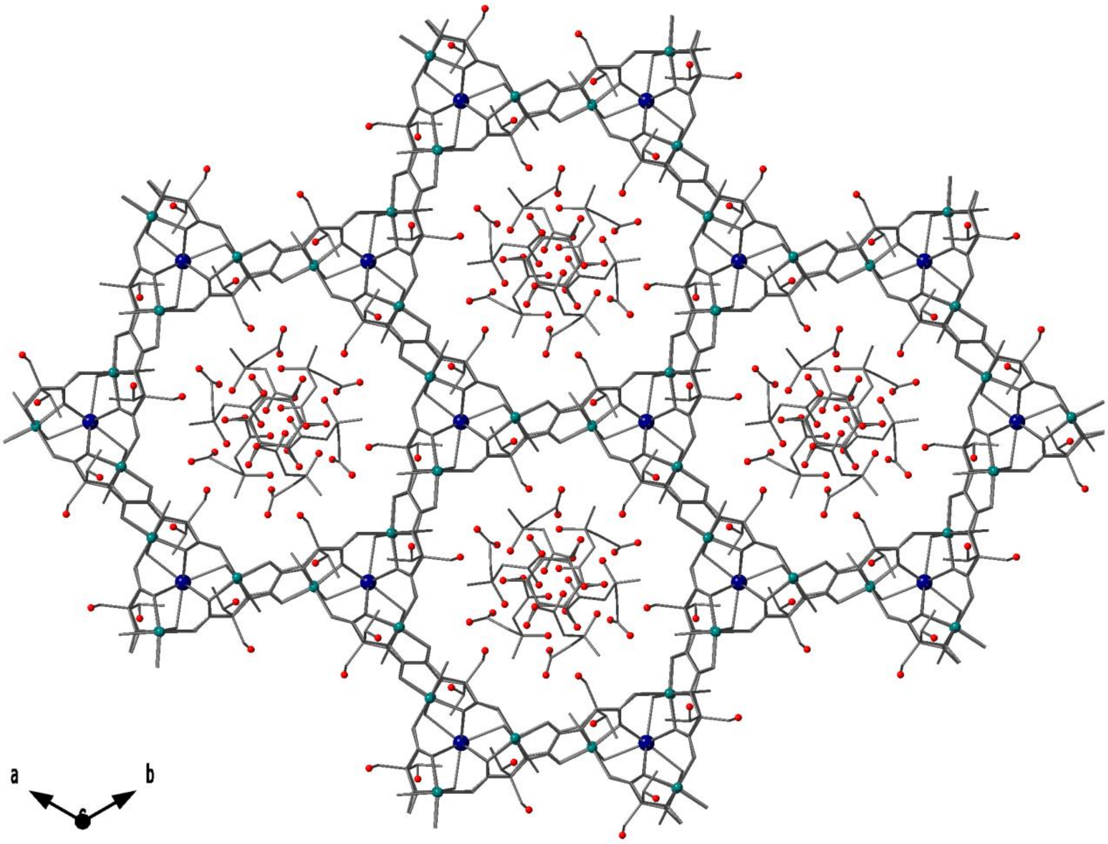


**Supplementary Fig. 17**. Perspective views along *c* crystallographic axis of crystal structure of **11a@2**. The 3D networks are depicted as grey sticks, with the only exception of copper(II), calcium(II) and serine residues oxygen atoms which are represented as green, blue and red sticks. Details of host-guest interactions are given in Supplementary Figs. 18 and 19 for the sake of clarity.

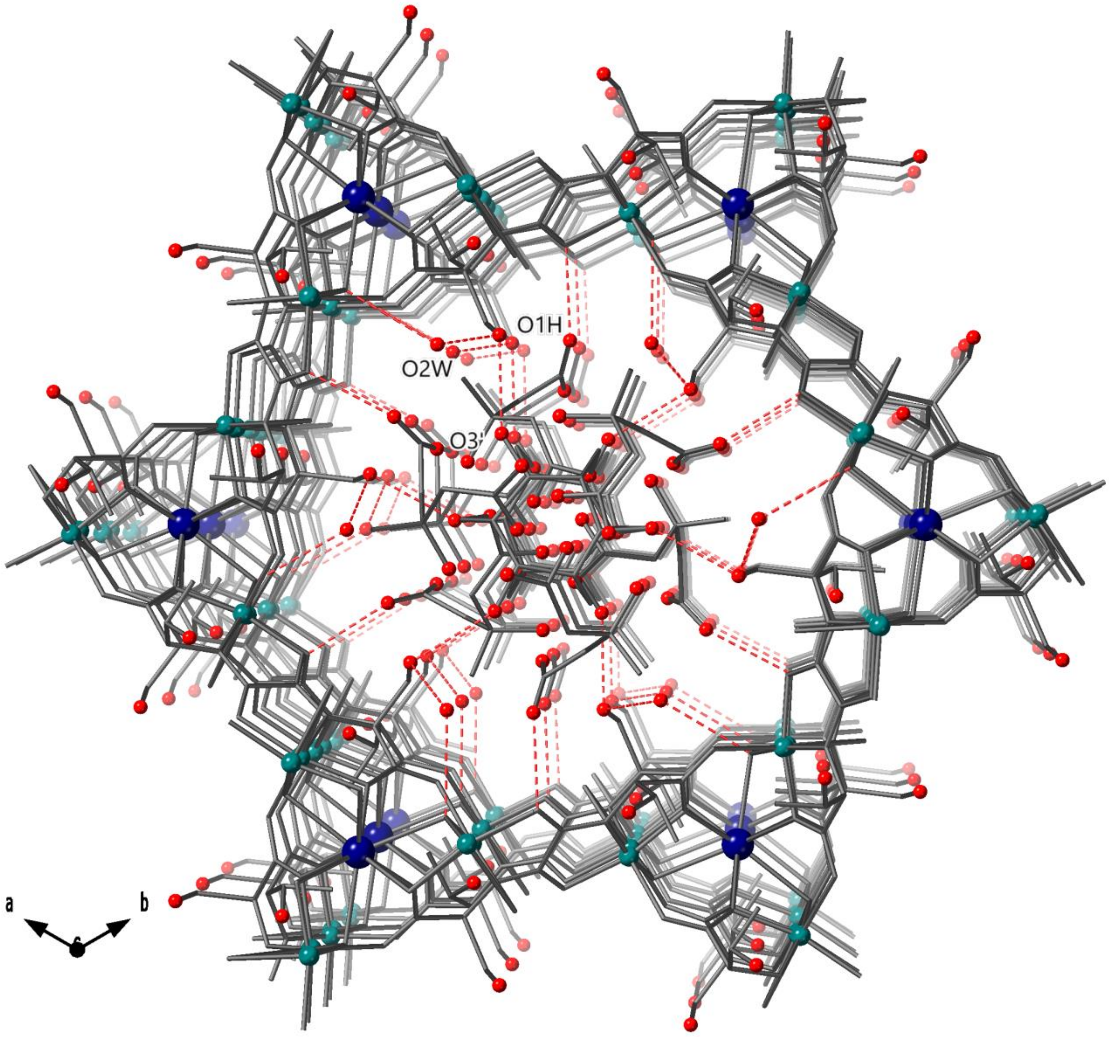


**Supplementary Fig. 18**. Perspective view of a single channel in crystal structure of **11a@2** emphasizing pores filled by guest molecules (grey sticks with the only exception of oxygen atoms, which are depicted as red spheres). The fragment of the 3D networks is also depicted as grey sticks, with the only exception of copper(II), calcium(II) and serine residues oxygen atoms which are represented as green, blue and red sticks. The H-bond interactions are depicted as red dashed lines. Lattice water molecules are not depicted for the sake of clarity except those "locked" being involved in the stabilization of the final serine residue configuration. This unique configuration turns more acid the alcoholic moiety. The crystallographically distinct serine arm, not so blocked, shows statistical disorder (see details of refinement in Crystallographic section).

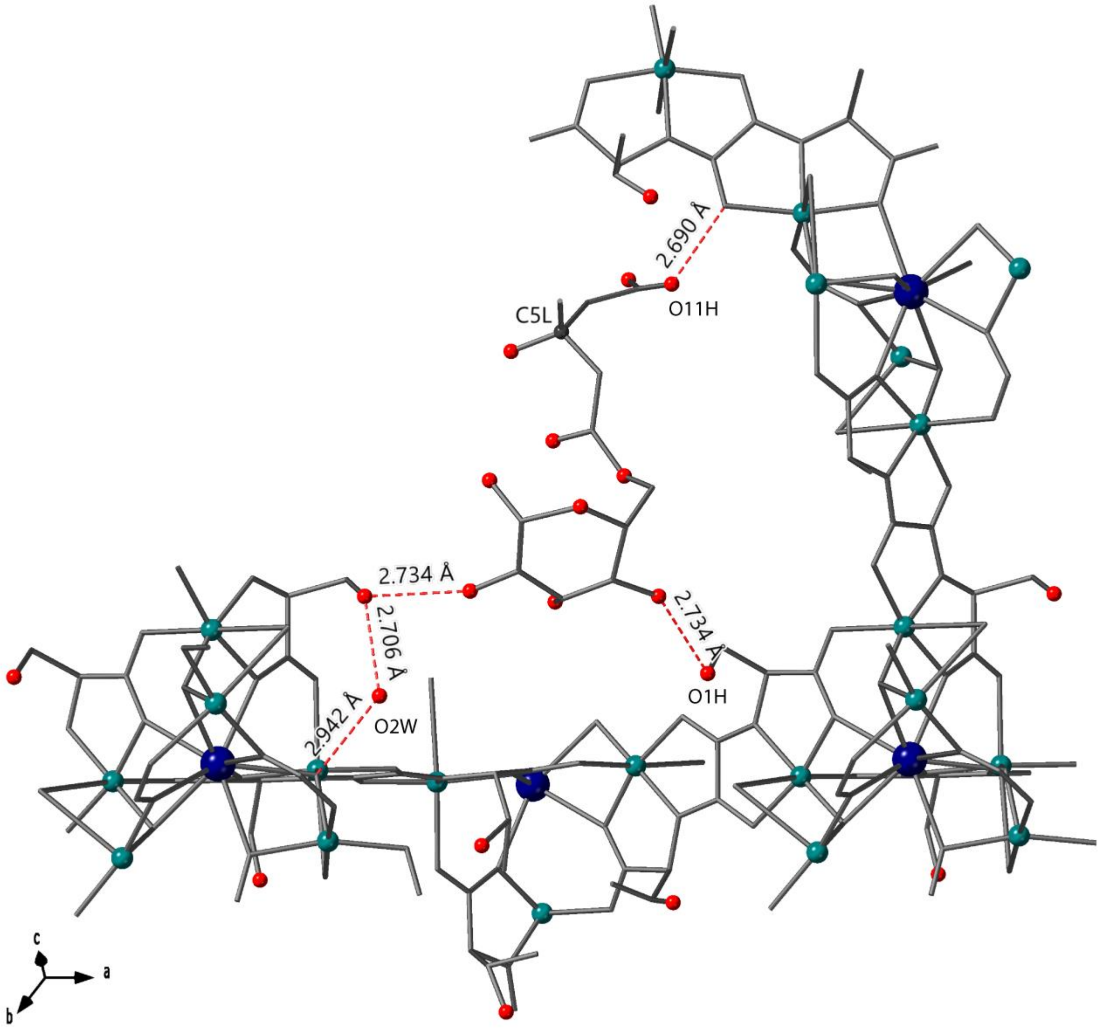


**Supplementary Fig. 19**. Details of host-guest interactions involving the chiral fragment **11a** packed within pores *via* strong and medium H-bonds (depicted as red dashed lines) involving hydroxyl groups of glucose moiety and terminal carboxylic groups linked to the net by serine arms and oxygen atoms from oxamate ligand. Atom colour code: All atoms from the fragment of the coordination network are represented as grey sticks, with the only exception of copper(II) (cyan spheres), calcium(II) (blue spheres) and oxygen atoms from serine residues and oxamate-oxygen atoms participating in the intermolecular interactions (red spheres). For the guest molecule, all oxygen atoms are depicted as red spheres whereas all carbon atoms – with the exception of C5L (grey sphere)– are depicted as grey sticks. Interacting free water molecules are represented as red spheres.

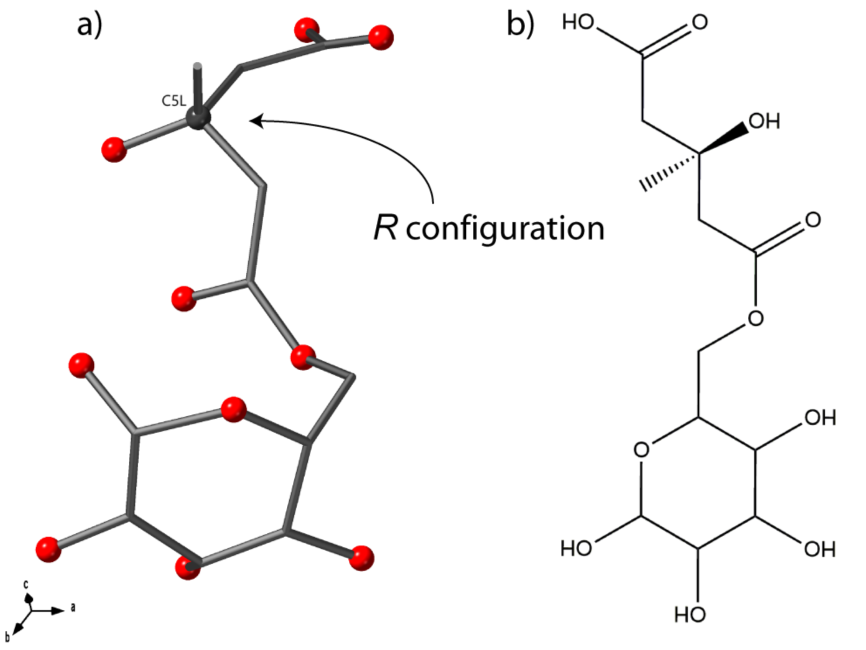


**Supplementary Fig. 20**. Crystal structure (a) and chemical draw (b) showing the absolute configuration of fragment **11a**.

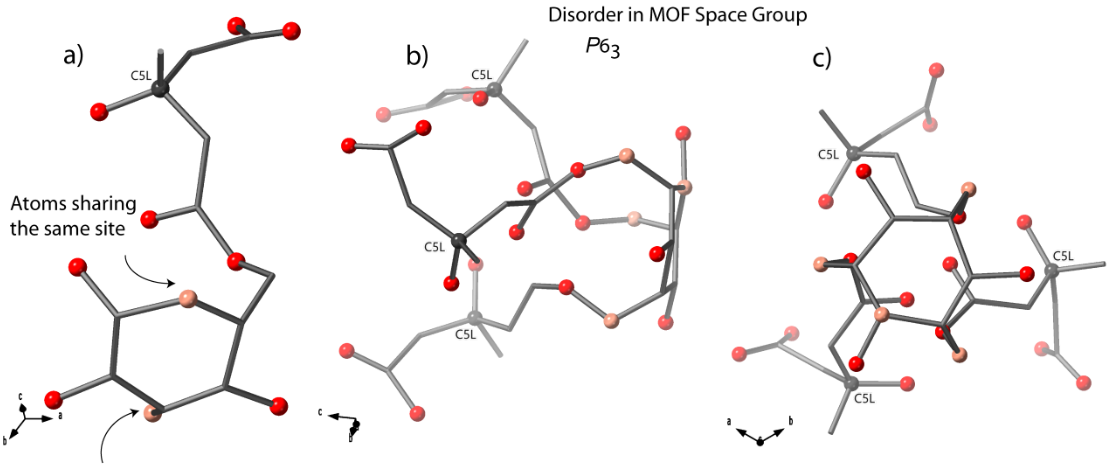


**Supplementary Fig. 21**. Details of crystallographic disorder of 6-O-(3′-hydroxy-3′-methylglutaryl)-glucopyranose), **11a** guest, in **11a@2** crystal structure. Crystal structure of **11a** where carbon and oxygen sites shared are depicted as pink spheres a). In MOF's Space Group three possible orientations of guest molecules are allowed (b-c).

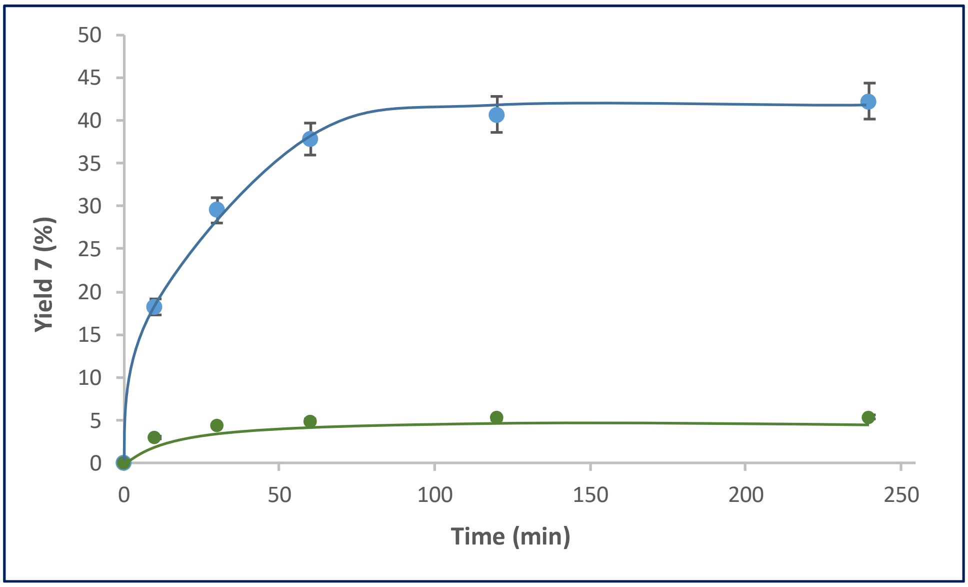


**Supplementary Fig. 22.** Comparison between MOF **2** (blue line) and building block $Cu_2^{II}$[(S,S)–serimox] (green line) as a catalyst for the hydrolysis of ketal **4**. Yields calculated by gas chromatography (GC) with *n*-dodecane as an external standard. See Supplementary Fig. 7 for reaction conditions. Error bars account for a 5% uncertaintity. Lines are a guide to the eye.

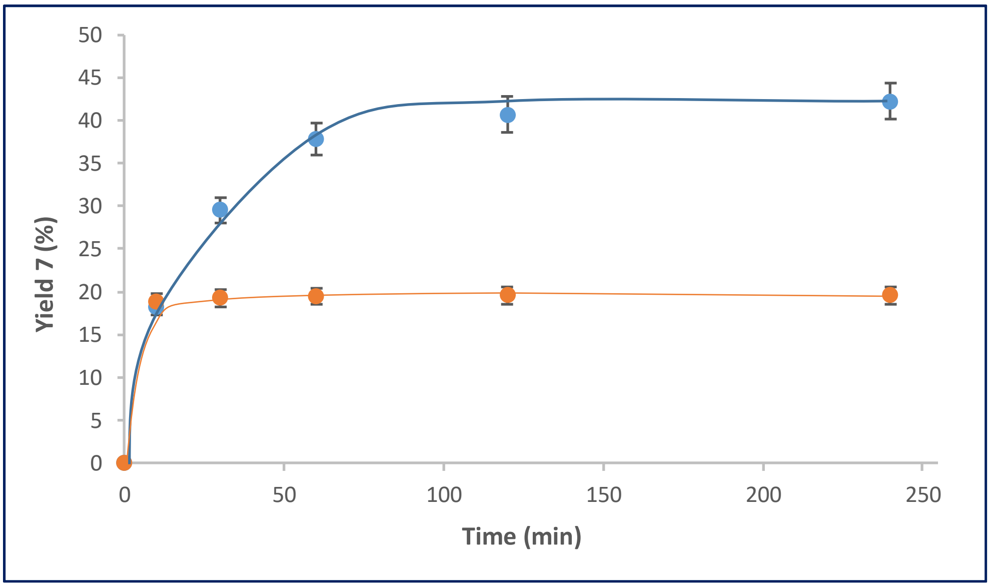


**Supplementary Fig. 23.** Filtration test for MOF **2**, without (blue line) and with filtering the solid catalyst after 15 min reaction time (orange line). See Supplementary Fig. 7 for reaction conditions. Error bars account for a 5% uncertaintity. Lines are a guide to the eye.

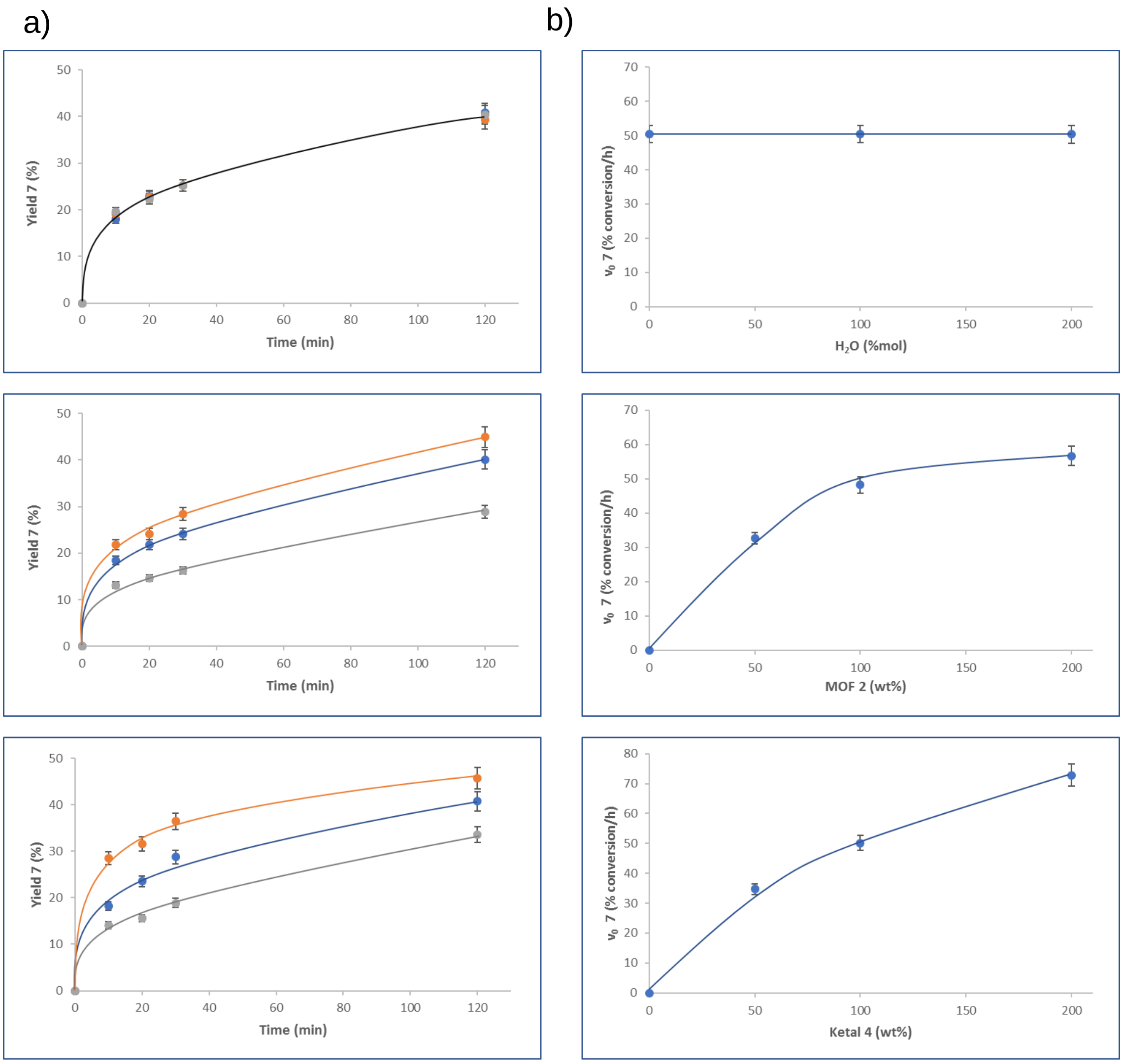


**Supplementary Fig. 24**. a) Kinetic experiments with increasing amounts (grey to blue to orange line) of MOF **2**, ketal **4** and water. b) Initial rates *vs.* reagent amounts, in order to stablish the reaction orders and the corresponding rate equation. Yields calculated by gas chromatography (GC) with *n*-dodecane as an external standard. See Figure S7 for reaction conditions. Error bars account for a 5% uncertaintity. Lines are a guide to the eye.

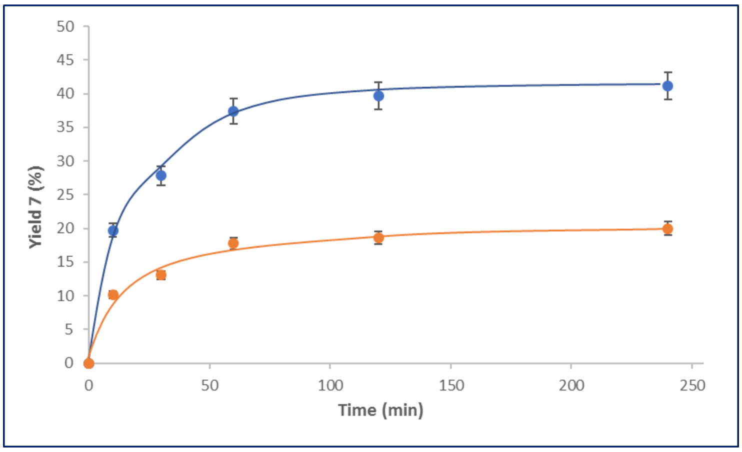


**Supplementary Fig. 25.** Comparison between kinetic points in the hydrolysis of ketal **4** with dehydrated (orange line) and original (wet, blue line) MOF **2** catalyst. Yields calculated by gas chromatography (GC) with *n*-dodecane as an external standard. Error bars account for a 5% uncertaintity. See Figure S7 for reaction conditions. Lines are a guide to the eye.

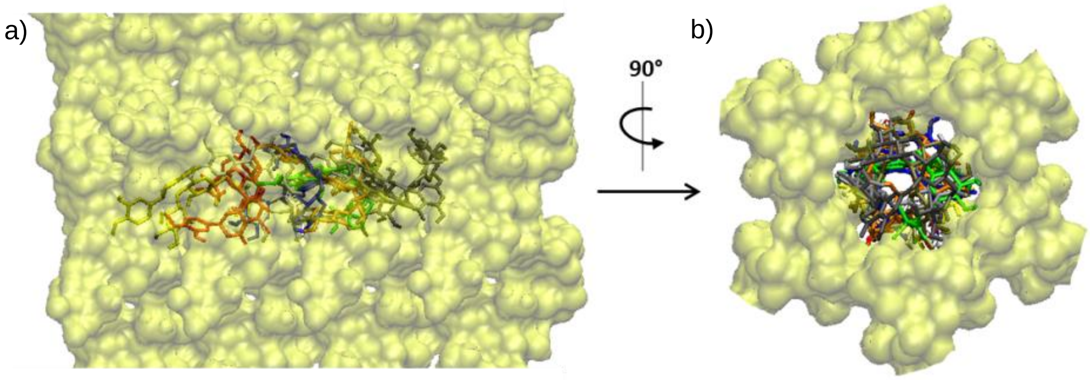


**Supplementary Fig. 26**. Side (a) and top (b) view of superimposed docked poses in the minimal unit of MOF **2**.

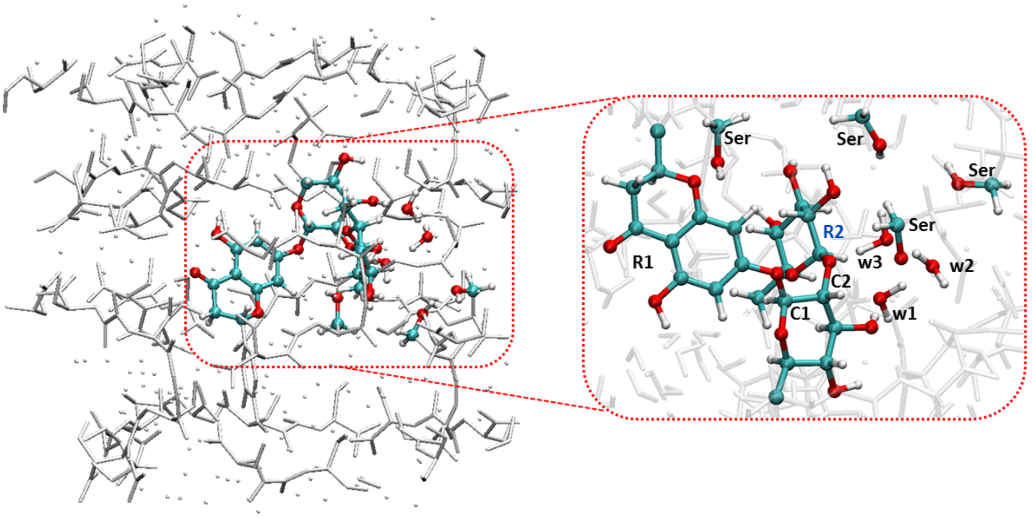


**Supplementary Fig. 27**. The model adopted in the QMMM investigation: on the left, is reported the whole system (1342 atoms) while in the red box the atoms retained in the QM region (76 atoms with one serine and 91 atoms with four serines).

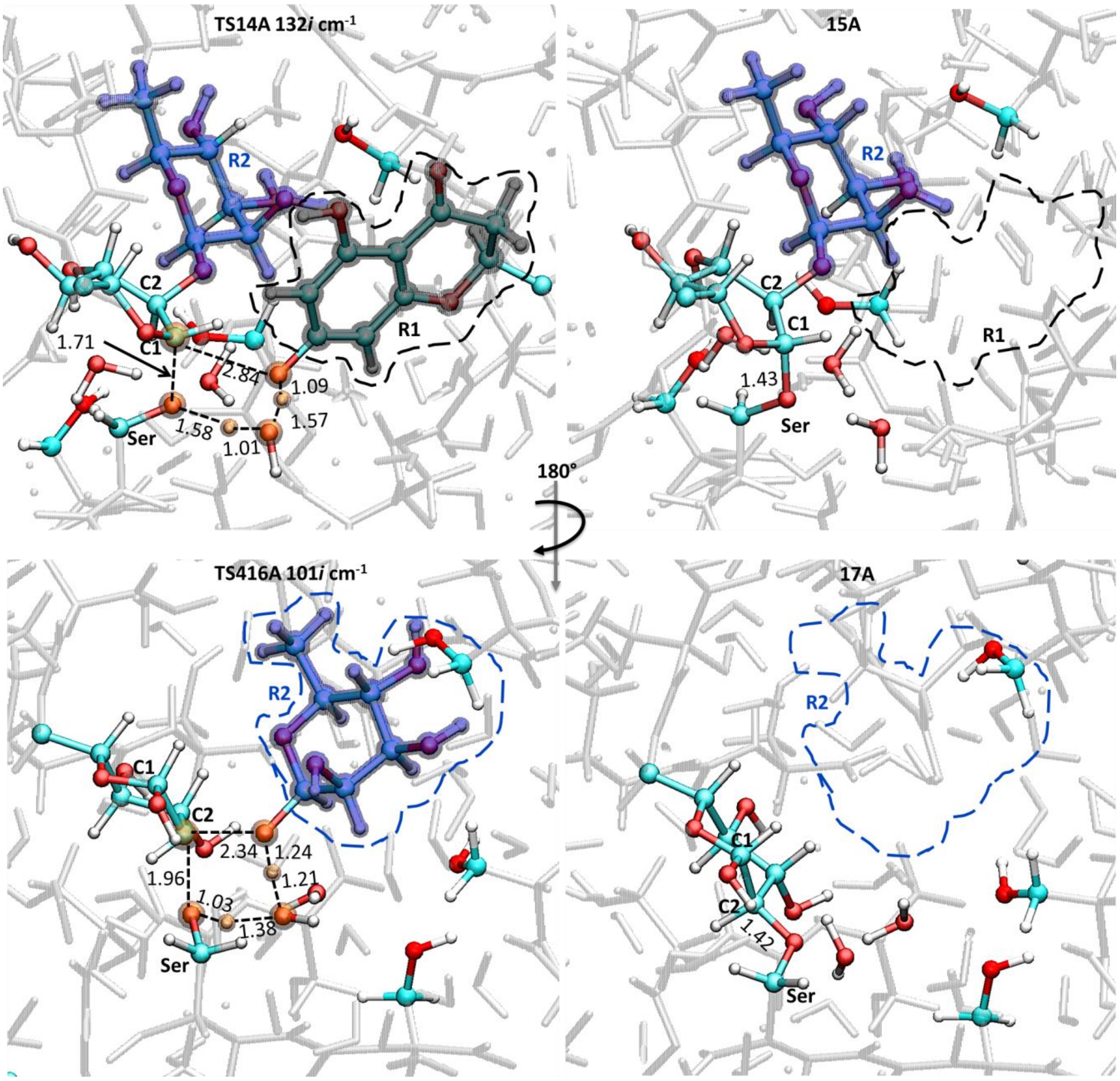


**Supplementary Fig. 28**. B3LYP/6-31G(d)|UFF optimized geometries of TS14A, 15A, TS16A and 17A stationary points. Imaginary frequencies ($cm^{-1}$) and main distances (Å) are also reported. For clarity are evidenced only the species participating in the reaction.

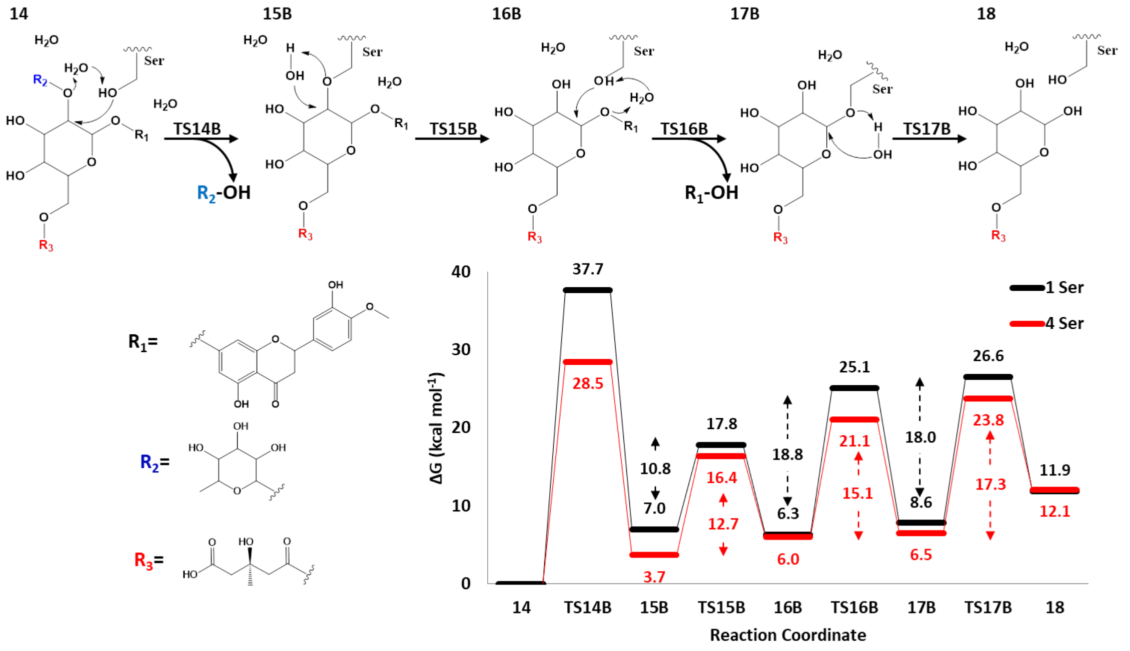


**Supplementary Fig. 29**. The catalytic mechanisms (top) followed in the hydrolysis reaction of **11** by MOF **2**, when C-O cleavage occurs initially to the R2 and successively on the R1, and related PES (right-bottom) calculated at B3LYP-D3/6-11+G(2d,2p)|UFF//B3LYP/6-31G(d)|UFF level of theory.

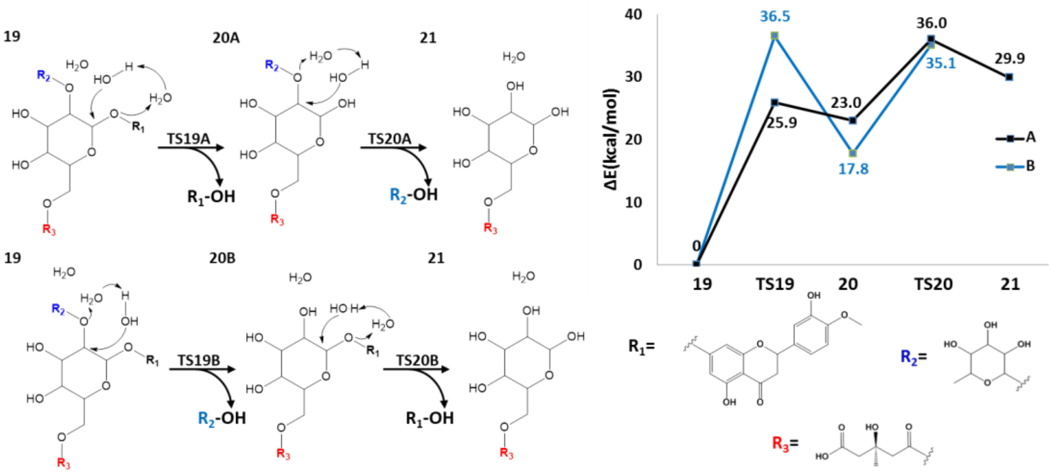


**Supplementary Fig. 30**. The reaction mechanisms (left) followed in the hydrolysis of **11** by three water molecules (in the absence of MOF **2**) and related PES (right calculated at B3LYP-D3/6-311+G(2d,2p)|UFF//B3LYP/6-31G(d)|UFF level of theory. The mechanism A describes the cleavage of R1 and R2

# Supplementary References


1. Mon, M. *et al.* Crystallographic snapshots of host–guest interactions in drugs@metal–organic frameworks: towards mimicking molecular recognition processes. *Mater. Horizons* **5**, 683–690 (2018).
2. Mosmann, T. Rapid colorimetric assay for cellular growth and survival: Application to proliferation and cytotoxicity assays. *J. Immunol. Methods* **65**, 55–63 (1983).
3. Di Donna, L. *et al.* Statin-like Principles of Bergamot Fruit ( Citrus bergamia ): Isolation of 3-Hydroxymethylglutaryl Flavonoid Glycosides. *J. Nat. Prod.* **72**, 1352–1354 (2009).
4. SAINT, version 6.45, Bruker Analytical X-ray Systems, Madison, W. SAINT, version 6.45, Bruker Analytical X-ray Systems, Madison, WI. (2003).
5. Sheldrick G.M. SADABS Program for Absorption Correction, version 2.10, Analytical X-ray Systems, Madison, W. SADABS Program for Absorption Correction, version 2.10, Analytical X-ray Systems, Madison, WI. (2003).
6. Sheldrick, G. M. A short history of SHELX. *Acta Crystallogr. A.* **64**, 112–22 (2008).
7. Sheldrick, G. M. Crystal structure refinement with SHELXL. *Acta Crystallogr. Sect. C Struct. Chem.* **71**, 3–8 (2015).
8. Spek, A. L. Structure validation in chemical crystallography. *Acta Crystallogr. Sect. D Biol. Crystallogr.* **65**, 148–155 (2009).
9. Spek, A. L. PLATON SQUEEZE: a tool for the calculation of the disordered solvent contribution to the calculated structure factors. *Acta Crystallogr. Sect. C Struct. Chem.* **71**, 9–18 (2015).
10. Farrugia, L. J. WinGX suite for small-molecule single-crystal crystallography. *J. Appl. Crystallogr.* **32**, 837–838 (1999).
11. Palmer, D. CRYSTAL MAKER, Cambridge University Technical Services, C. CRYSTAL MAKER, Cambridge University Technical Services, Cambridge. (1996).
12. Huang, F.-C. *et al.* Preparation of (R)- and (S)-mevalonic acids. *J. Am. Chem. Soc.* **97**, 4144–4145 (1975).
13. Wilson, W. K., Baca, S. B., Barber, Y. J., Scallen, T. J. & Morrow, C. J. Enantioselective hydrolysis of 3-hydroxy-3-methylalkanoic acid esters with pig liver esterase. *J. Org. Chem.* **48**, 3960–3966 (1983).
14. Trott, O. & Olson, A. J. AutoDock Vina: Improving the speed and accuracy of docking with a new scoring function, efficient optimization, and multithreading. *J. Comput. Chem.* **31**, 455–461 (2010).
15. Frisch, M. J., Trucks, G. W., Schlegel, H. B., Scuseria, G. E., Robb, M. A., Cheeseman, J. R., Scalmani, G., Barone, V., Petersson, G. A., Nakatsuji, H., Li, X., Caricato, M., Marenich, A., Bloino, J., Janesko, B. G., Gomperts, R., Mennucci, B., Hratchian, H. P., Ortiz, J. V., Izmaylov, A. F., Sonnenberg, J. L., Williams-Young, D., Ding, F., Lipparini, F., Egidi, F., Goings, J., Peng, B., Petrone, A., Henderson, T., Ranasinghe, D., Zakrzewski, V. G., Gao, J., Rega, N., Zheng, G., Liang, W., Hada, M., Ehara, M., Toyota, K., Fukuda, R., Hasegawa, J., Ishida, M., Nakajima, T., Honda, Y., Kitao, O., Nakai, H., Vreven, T., Throssell, K., Montgomery, J. A., Jr., Peralta, J. E., Ogliaro, F., Bearpark, M., Heyd, J. J., Brothers, E., Kudin, K. N., Staroverov, V. N., Keith, T., Kobayashi, R., Normand, J., Raghavachari, K., Rendell, A., Burant, J. C., Iyengar, S. S., Tomasi, J., Cossi, M., Millam, J. M., Klene, M., Adamo, C., Cammi, R., Ochterski, J. W., Martin, R. L., Morokuma, K., Farkas, O., Foresman, J. B., Fox, D. J. Gaussian 09, Revision C.01; Gaussian 16, Revision A.03, Gaussian, Inc., Wallingford CT, Gaussian 09, Revision D.01 (2016).
16. Lee, C., Yang, W. & Parr, R. G. Development of the Colle-Salvetti correlation-energy formula into a functional of the electron density. *Phys. Rev. B* **37**, 785–789 (1988).
17. Becke, A. D. Density-functional thermochemistry. III. The role of exact exchange. *J. Chem. Phys.* **98**, 5648–5652 (1993).
18. Svensson, M. *et al.* ONIOM: A Multilayered Integrated MO + MM Method for Geometry Optimizations and Single Point Energy Predictions. A Test for Diels−Alder Reactions and Pt(P(t -Bu)3)$^{2+}$ $H_2$ Oxidative Addition. *J. Phys. Chem.* **100**, 19357–19363 (1996).
19. Vreven, T. *et al.* Combining Quantum Mechanics Methods with Molecular Mechanics Methods in ONIOM. *J. Chem. Theory Comput.* **2**, 815–826 (2006).
20. Grimme, S., Ehrlich, S. & Goerigk, L. Effect of the damping function in dispersion corrected density functional theory. *J. Comput. Chem.* **32**, 1456–1465 (2011).
